# Telomere damage promotes vascular smooth muscle cell senescence and immune cell recruitment after vessel injury

**DOI:** 10.1038/s42003-021-02123-z

**Published:** 2021-05-21

**Authors:** Anna K. Uryga, Mandy O. J. Grootaert, Abel M. Garrido, Sebnem Oc, Kirsty Foote, Joel Chappell, Alison Finigan, Francesca Rossiello, Fabrizio d’Adda di Fagagna, Dimitra Aravani, Helle F. Jorgensen, Martin R. Bennett

**Affiliations:** 1 Division of Cardiovascular Medicine, University of Cambridge, Addenbrooke’s Centre for Clinical Investigation, Addenbrooke’s Hospital, Cambridge, UK; 2 FIRC Institute of Molecular Oncology Foundation, IFOM Foundation, Milan, Italy; 3 Istituto di Genetica Molecolare, CNR-Consiglio Nazionale delle Ricerche, Pavia, Italy

**Keywords:** Mechanisms of disease, Senescence

## Abstract

Accumulation of vascular smooth muscle cells (VSMCs) is a hallmark of multiple vascular pathologies, including following neointimal formation after injury and atherosclerosis. However, human VSMCs in advanced atherosclerotic lesions show reduced cell proliferation, extensive and persistent DNA damage, and features of premature cell senescence. Here, we report that stress-induced premature senescence (SIPS) and stable expression of a telomeric repeat-binding factor 2 protein mutant (TRF2^T188A^) induce senescence of human VSMCs, associated with persistent telomeric DNA damage. VSMC senescence is associated with formation of micronuclei, activation of *cGAS-STING* cytoplasmic sensing, and induction of multiple pro-inflammatory cytokines. VSMC-specific TRF2^T188A^ expression in a multicolor clonal VSMC-tracking mouse model shows no change in VSMC clonal patches after injury, but an increase in neointima formation, outward remodeling, senescence and immune/inflammatory cell infiltration or retention. We suggest that persistent telomere damage in VSMCs inducing cell senescence has a major role in driving persistent inflammation in vascular disease.

## Abstract (web summary)

Anna Uryga and Mandy Grootaert et al. combine cell culture and animal models to examine how senescence of human vascular smooth muscle cells (VSMCs) and persistent telomere damage drive inflammation. Their results suggest that telomere injury can be the primary cause of premature senescence in VSMCs, and that DNA damage can be a major cause of persistent inflammation in vascular disease.

## Introduction

Injury to human arteries induces proliferation and migration of vascular smooth muscle cells (VSMCs) to form a neointima, whereas VSMC accumulation is also a major feature of the atherosclerotic plaque intima. However, in both the neointima after injury and plaques, only a small subset of VSMCs proliferate to form monoclonal or oligoclonal patches within lesions^1,2^. Extensive proliferation of a VSMC subset may result in cellular senescence due to the exhaustion of replicative potential, in large part owing to damage to telomeres. A range of extrinsic and intrinsic stimuli also induce DNA damage and cell senescence, termed “stress-induced premature senescence” (SIPS)^3^. We have shown previously that telomere length is reduced by oxidative stress in human VSMCs^4,5^, and human plaque VSMCs show extensive and persistent DNA damage, including reduced telomere signals in vivo^4^ and premature senescence in vitro, associated with reduced binding of specific telomere proteins to telomere DNA^6^. Similarly, VSMCs forming the fibrous caps of mouse plaques show the highest numbers of divisions^7^, suggesting that VSMC senescence may be a major driver of both intimal and neointimal formation. However, whether VSMC senescence promotes intimal/neointimal formation is unclear, particularly whether it is due primarily to the loss of VSMC replicative function or secondary consequences in senescent VSMCs, the mechanisms involved, and whether VSMC senescence promotes clonality.

Cells have tightly coordinated pathways to sense DNA damage and promote its repair, termed the “DNA damage response“ (DDR). Most DNA damage is repairable, but destabilization of telomere integrity owing to repeated cell division or loss of key telomere binding proteins induces a persistent DDR^8–10^ and leads to cellular senescence^11–13^. In response to a persistent DDR, senescent cells undergo profound cellular and molecular changes, including secretion of inflammatory cytokines, chemokines, growth factors, and proteases, termed the senescence-associated secretory phenotype (SASP)^14–16^. SASP components reinforce the senescent phenotype and also affect the tissue microenvironment in a cell non-autonomous fashion^17–21^, such that few senescent cells can have large effects on tissue structure. Although DNA damage appears to be required for the SASP, the precise mechanisms coupling the DDR to the SASP remain elusive, particularly in vascular cells. Senescent cells have been reported to release nuclear DNA fragments into the cytoplasm^22–25^ that activate the cyclic GMP-AMP (cGAMP) synthase (*cGAS*) cytoplasmic DNA sensor. Typically, binding of DNA by cGAS catalyzes the synthesis of second messenger cyclic cGAMP, which in turn activates the adaptor protein STING and its downstream mediators TANK binding kinase 1 (TBK1) and nuclear factor kappa-light-chain-enhancer of activated *B* cells (NF-κB), leading to upregulation of pro-inflammatory genes^26^. However, there is wide variability of *cGAS/STING* signaling between different senescent cell types, and a number of different pathways link DNA damage and induction of pro-inflammatory cytokines.

We examined DNA damage following SIPS or more precise telomere damage. Telomere stability is maintained by a number of telomere-associated proteins known as the shelterin complex, which protects telomere ends from being recognized as double-strand breaks (DSBs)^27^. In particular, telomeric repeat-binding factor 2 (TRF2) is a key shelterin protein responsible for telomere protection, as conditional deletion or overexpression of mutated forms results in dysfunctional telomeres, telomere fusions, and cell senescence^6,8,28^.

We show that both SIPS and expression of a mutant TRF2 protein (TRF2^T188A^) induce persistent telomere damage and senescence in VSMCs, and a pro-inflammatory phenotype, which is reliant on *cGAS*-*STING* signaling but restricted to a subset of pro-inflammatory genes. Persistent telomere damage in VSMCs in vivo induces a marked pro-inflammatory/immune cell infiltrate with increased neointimal formation and vessel remodeling, but no change in clonality of VSMCs. Our results indicate that VSMC senescence may exert its major effects through inflammation rather than impaired proliferation, and persistent telomere damage may be an important driver of the chronic inflammation seen in vascular disease.

## Results

### Telomere damage is persistent in human VSMC senescence

**Fig. 1:**
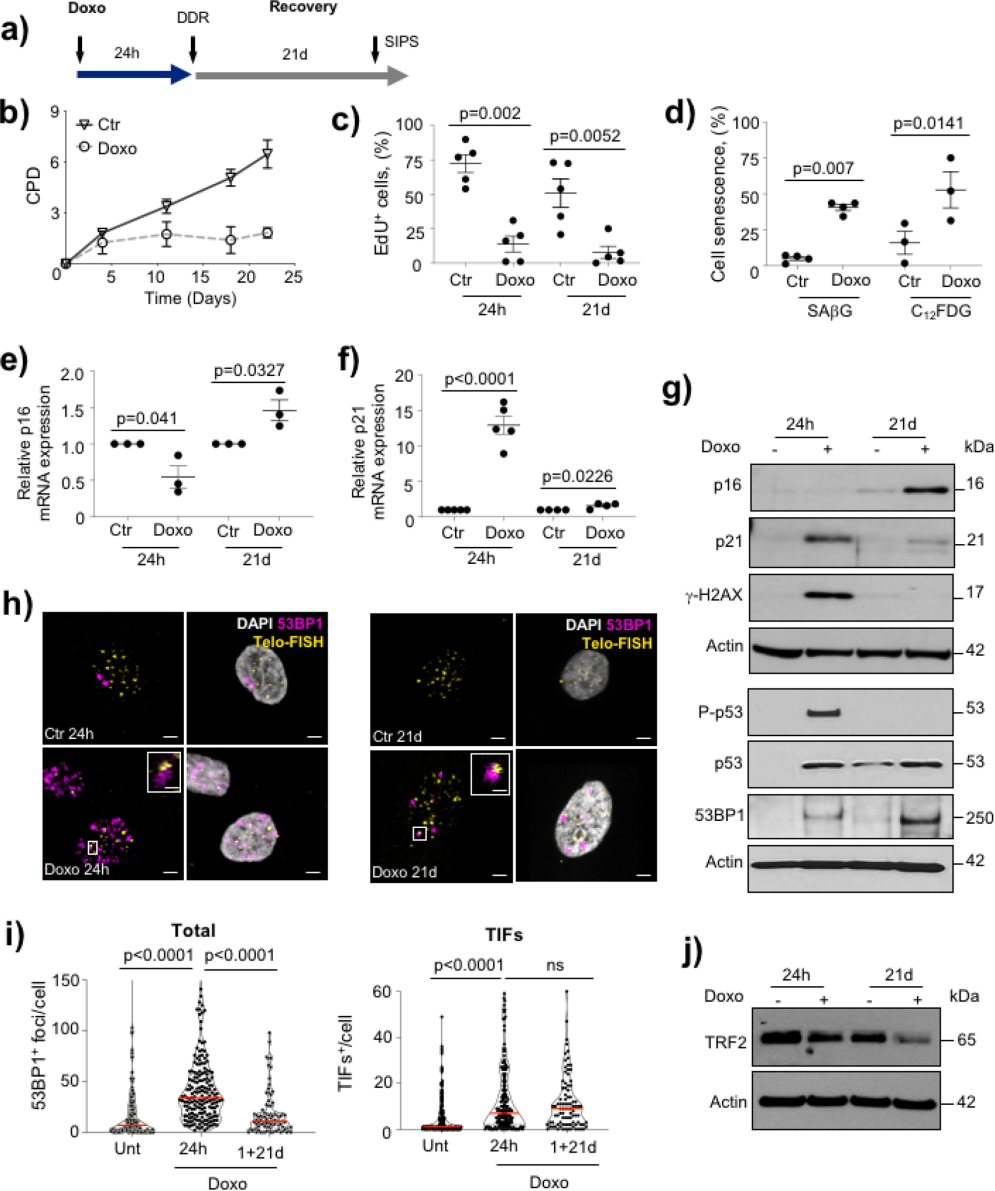
Stress-induced premature senescence induces persistent telomere damage in human VSMCs. **a** Experimental timeline of human VSMCs treated with 500 nM doxorubicin for 24 h to induce either acute DNA damage or recovered for 21d to induce SIPS. **b** Cumulative population doublings (CPD) following doxo 1d + 21d or 1d + 21d controls. **c** % EdU^+^ cells following doxo 24 h or 1d + 21d, or 24 h or 1d + 21d controls. **d** % SAβG or C_12_FDG-positive cells following doxo 1d + 21d or 1d + 21d controls. **e**–**f** qPCR for *p16* and *p21* and **g** Western blotting for p16, p21, γ-H2AX, phospho-p53, p53, and 53BP1 in VSMCs following doxo 24 h or 1d + 21d, or 24 h or 1d + 21d controls. **h** 53BP1 immuno-Telo-FISH DAPI (white), telo-FISH (yellow), and 53BP1 (magenta). Magnified images show colocalizing foci. Scale bar = 5 μm and 1.2 μm (inset). **i** Mean number of 53BP1 (total) foci or TIFs following doxo 24 h or 1d + 21d or untreated control cells (Unt). More than 30 cells were analyzed/condition per experiment. **j** Western blot for TRF2 in VSMCs following doxo 24 h or 1d + 21d, or 24 h, or 1d + 21d controls. Data shown are means±SEM, *n* = 3–5 independent experiments, ns-non significant (*p* > 0.05), two-tailed Student’s *t* test or one-way ANOVA, with Dunnett’s correction test.

To study the consequences of either acute genomic DNA damage or SIPS, respectively, we harvested human VSMCs after treatment with the chemotherapeutic agent doxorubicin for 24 h (dox24h), or for 1 day followed by 21 days recovery (dox1d + 21d) together with respective untreated controls (control 24 h and control 21d) (Fig. 1a). Compared with control cells, dox1d + 21d cells showed no increase in cumulative population doublings (CPDs) after day 5, suggesting persistent cell cycle arrest (Fig. 1b). Both dox24h and dox1d + 21d-treated cells showed reduced cell proliferation compared with their respective controls (Fig. 1c), and senescence-associated β galactosidase (SAβG) activity was increased in dox1d + 21d-treated cells measured using X-gal staining and a C_12_FDG-based fluorescent substrate (Fig. 1d). Senescence induced by dox1d + 21d, but not acute DNA damage, induced mRNA and protein expression of the cyclin-dependent kinase inhibitor *p16*^*ink4a*^, whereas *p21*^*cip1/waf1*^ was only upregulated upon acute DNA damage and normalized by 21d recovery (Fig. 1e–g). SIPS also increased protein expression of both p53 and 53BP1, a DDR marker that is particularly upregulated during non-homologous end joining (NHEJ) (Fig. 1g). Similar to p21^cip1/waf1^, phosphorylated p53 and γ-H2AX that are robust but more generalized DDR markers were only induced upon acute DNA damage (Fig. 1g).

Acute doxorubicin treatment generates widespread DNA damage and focal accumulation of DDR proteins in the nucleus (DDR foci) that are efficiently repaired over time. However, telomeric DNA sequences generally show impaired repair, resulting in the accumulation of telomere dysfunction-induced foci (TIFs). We used telomere immunofluorescence in situ hybridization (telo-FISH) to quantify the number of telomere signals that colocalize with the DDR protein 53BP1, as a proportion of the total number of 53BP1 foci. Doxorubicin increased the number of 53BP1 foci and TIFs at 24 h; however, although total 53BP1 foci declined to baseline after 21d recovery, similar to γ-H2AX expression, TIFs did not (Fig. 1h, i). Interestingly, SIPS was also associated with reduced expression of the TRF2 protein (Fig. 1j), indicating that uncapped telomeres could contribute to the DDR in VSMC senescence. These data suggest that while the majority of DNA damage can be repaired in VSMCs, SIPS is characterized by persistent telomere damage and DDR signaling associated with reduced TRF2 expression in VSMCs.

### TRF2^T188A^ induces a senescence-like phenotype via telomere damage

**Fig. 2:**
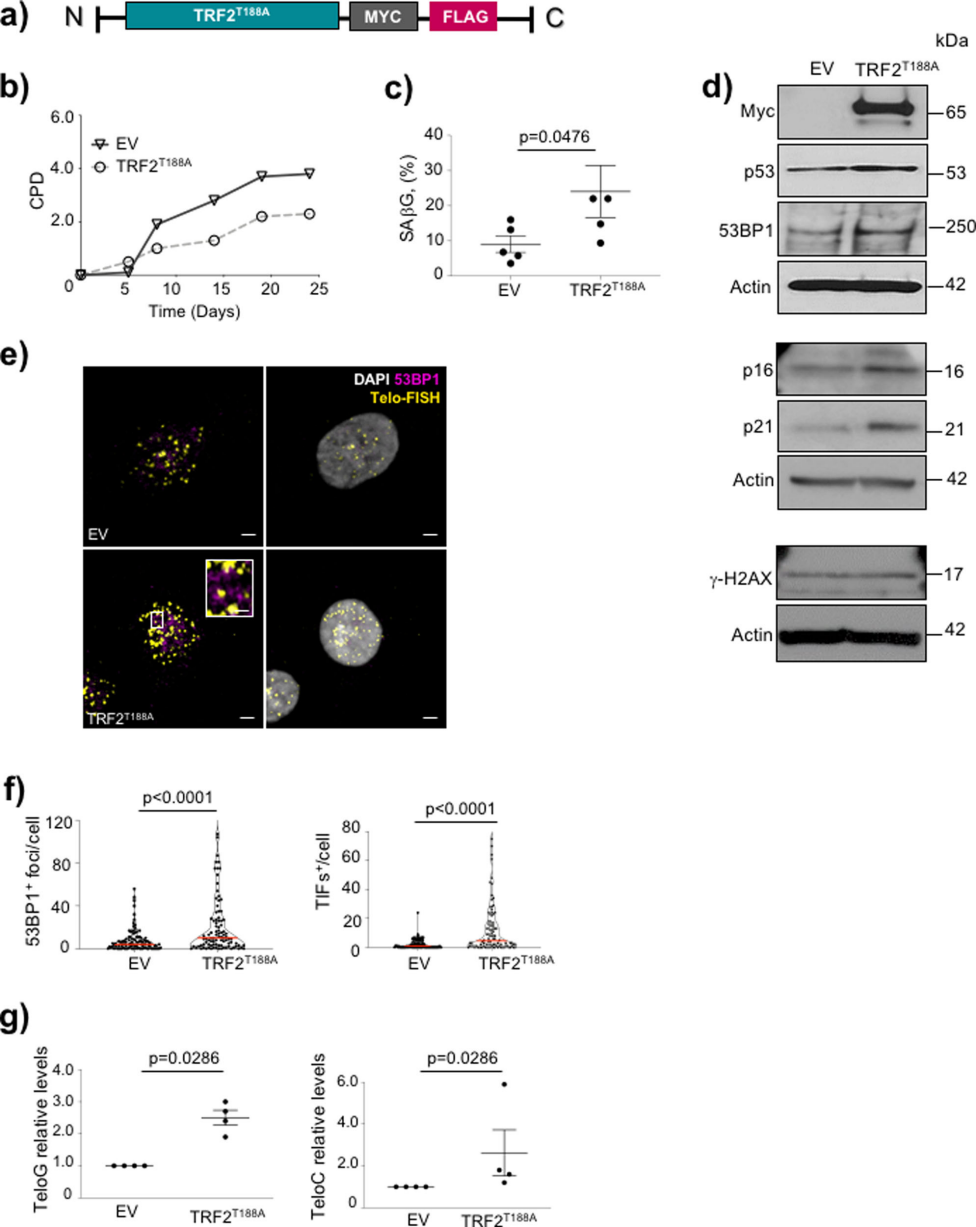
Telomere-specific DNA damage promotes senescence-like phenotype. **a** Schematic of TRF2^T188A^ vector construct. **b** Cumulative population doublings (CPDs) over 25 days in empty vector (EV) or TRF2^T188A^ VSMCs. **c** % SAβG-positive cells in empty vector (EV) or TRF2^T188A^ VSMCs. **d** Western blot for c-Myc, p53, 53BP1, p16, p21, and γ-H2AX in EV or TRF2^T188A^ VSMCs at 21d after transfection. **e** 53BP1 immuno-Telo-FISH in EV or TRF2^T188A^ VSMCs; DAPI (white), telo-FISH (yellow), and 53BP1 (magenta). Magnified images show colocalizing foci. Scale bar = 5 μm and 1.5 μm (inset). **f** Mean number of 53BP1 (total) or TIF-positive foci in EV and TRF2^T188A^ cells. More than 30 cells were analyzed/condition per experiment. **g** Strand-specific RT-QPCR for dilncRNAs (*TeloC* and *TeloG*) in empty EV or TRF2^T188A^ VSMCs. Data are means±SEM, *n* = 3–5 independent experiments, Mann–Whitney *U* test.

We have previously shown that expression of the TRF2^T188A^ point mutant induces DNA damage and VSMC senescence in mouse VSMCs^6^. TRF2^T188A^ expression was associated with defective DDR signaling after oxidative DNA damage, and with marked damage to chromosomal ends^6^, suggesting that TRF2^T188A^ induces VSMC senescence predominantly via telomere damage. To study the effect of persistent telomere damage in human VSMCs, we generated lentiviruses with either an empty vector (EV) or expressing a tagged TRF2^T188A^ protein (Fig. 2a). Compared with EV cells, human TRF2^T188A^ VSMCs achieved fewer CPDs (Fig. 2b), had an increased percentage of cells showing SAβG activity (Fig. 2c, Supplementary Figure 1), and increased expression of p53, p16, and p21, but not γ-H2AX (Fig. 2d), indicating cell senescence. TRF2^T188A^ VSMCs also showed increased expression of 53BP1 (Fig. 2d) and an increase in the total number of 53BP1 foci and TIFs, but with the majority of 53BP1 foci being TIFs (Fig. 2e, f). Telomere damage also results in the generation of DNA damage-induced long non-coding RNA (dilncRNA) transcribed exclusively from telomeres on both strands (*teloG* and *teloC*), which can be used to determine the presence of telomeric damage^29^. Expression of both the dilncRNAs *teloG* and *teloC* were significantly increased in TRF2^T188A^ vs. EV VSMCs (Fig. 2g), further confirming an active telomere DDR in TRF2^T188A^ cells.

### Human VSMC senescence induces multiple genes associated with the SASP and telomere damage/repair

**Fig. 3:**
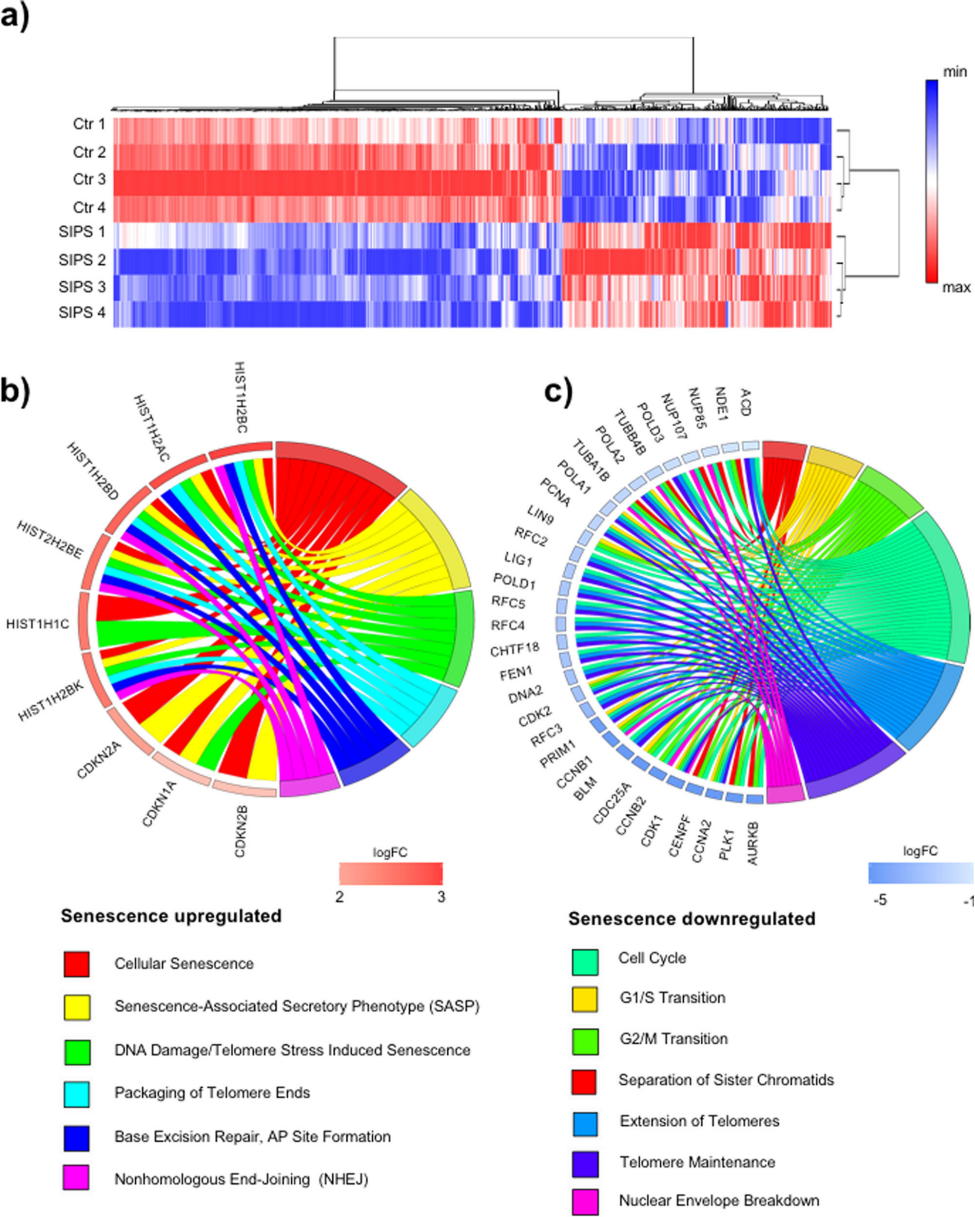
SIPS induces genes associated with the telomere damage and SASP. **a** Hierarchical clustering and heatmap visualization of up- and downregulated genes (FDR < 0.05) after dox1d + 21d treatment (SIPS1-4) compared with control primary hVSMC lines (Ctr1–4). The scale bar represents gene expression difference between the maximum and minimum values for each gene of a log_2_-scale. **b** Chord plot for selected gene ontogeny (GO) term pathways that were significantly associated (adjusted *p* value < 0.05) with genes upregulated in SIPS, along with their gene members (left) within the upregulated gene set. **c** Chord plot for selected GO term pathways that were significantly associated (adjusted *p* value < 0.05) with genes downregulated in SIPS, along with their gene members (left) within the downregulated gene set, filtered for involvement in at least three pathways.

Having established that both SIPS and TRF2^T188A^ expression result in persistent telomere damage, we used bulk RNA sequencing to identify the transcriptional signatures of senescent vs actively replicating cells, and thereby reveal pathways associated with human VSMC senescence. Human primary aortic VSMC cultures from four separate donors (two male, two female, average age 65 y) were treated with dox1d + 21d (SIPS) and compared with the same four lines cultured in parallel for 22d without doxorubicin. Hierarchical clustering and heatmaps of differentially expressed genes showed very similar patterns within each group, but marked differences between SIPS and control cells (Fig. 3a, Supplementary Data 1). Reactome pathway analysis showed enrichment for cellular senescence and the SASP (including cyclin-dependent kinase inhibitor 2 A (*cdkn2a*) encoding p16^ink4a^ and p14^ARF^) and DNA damage/telomere stress-induced senescence in genes expressed higher by SIPS vs. control cells (Fig. 3b and Supplementary Data 2). Downregulated genes in SIPS vs control cells were significantly associated with the cell cycle, telomere maintenance such as DNA replication helicase/nuclease 2 (*dna2*) and DNA ligase 1 (*lig1*), and nuclear envelope turnover (Fig. 3c and Supplementary Data 2). This analysis indicates that the senescence of human VSMCs is associated with a SASP, telomere damage, DNA repair, and possibly changes in nuclear envelope structure.

### TRF2^T188A^ promotes inflammation in VSMCs through activation of the cGAS-STING-TBK1 pathway

**Fig. 4:**
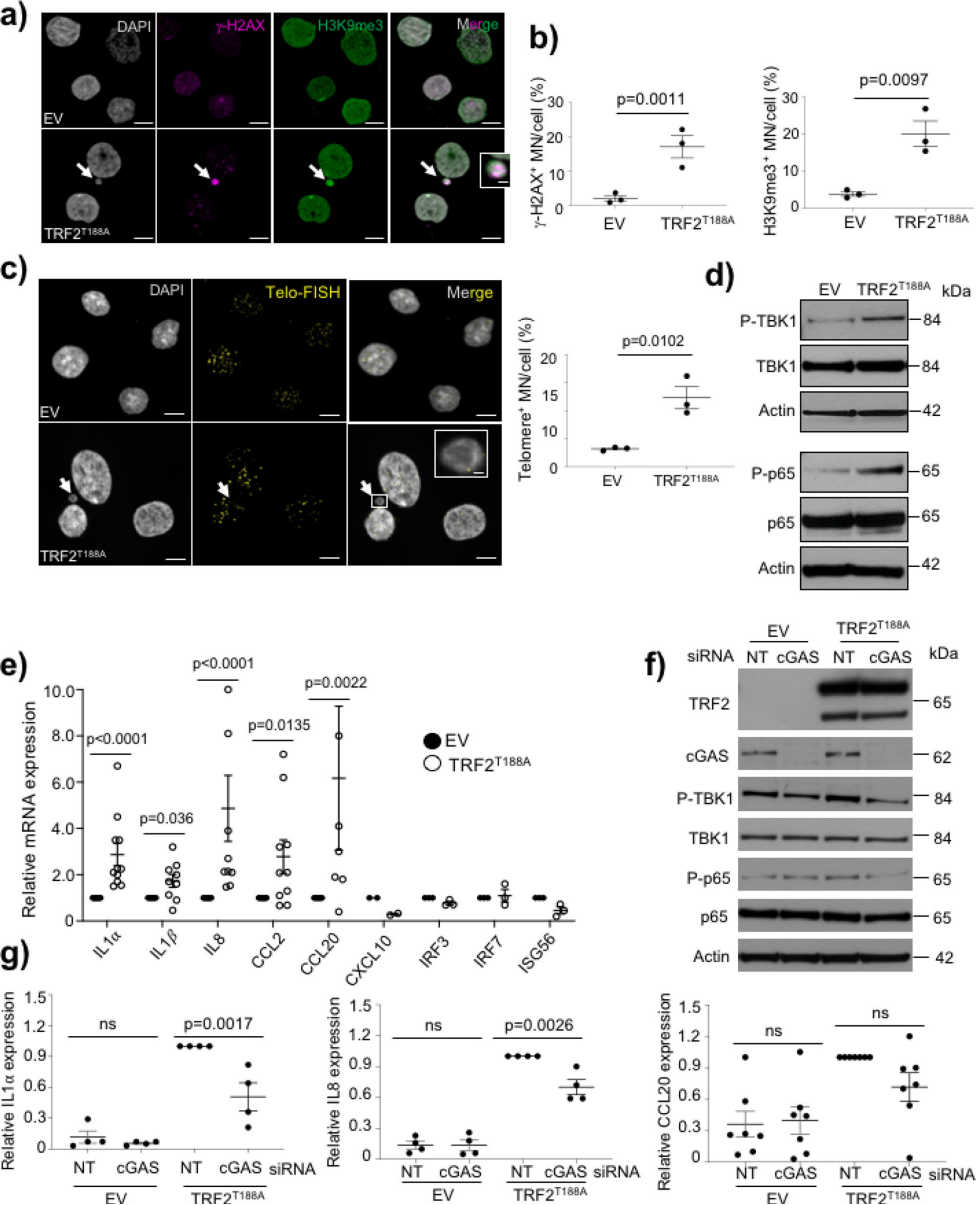
Telomere-specific DNA damage promotes SASP partly via *cGAS-STING-TBK1* pathway. **a** Immunocytochemistry and **b** quantification of γ-H2AX^+^, H3K9me3^+^, or Telomere^+^ micronuclei (MN—white arrows) in EV and TRF2^T188A^ VSMCs. DAPI (white); H3K9me3 (green), and γ-H2AX (magenta). Magnified upper panel shows H3K9me3 and γ-H2AX colocalizing with MN. Scale bar = 10 μm and 1 μm (inset). **c** Human VSMCs containing the empty vector (EV) or expressing TRF2^T188A^ stained with DAPI (white) and telo-FISH (yellow). Magnified outlined region shows telo-FISH colocalizing with MN. Scale bar = 10 μm and 2 μm (inset). **d** Western blot for TBK1 and p65 NF-κB and their phosphorylated forms in EV and TRF2^T188A^ cells. **e** Relative mRNA expression of selected cytokines in EV or TRF2^T188A^ VSMCs. **f** Western blot for cGAS, TBK1, p65 NF-κB, and their phosphorylated forms in EV and TRF2^T188A^ cells transfected with non-targeting (NT)- or *cGAS*-siRNA. **g** Relative mRNA expression of *IL1α, IL8,* and *CCL20* in EV or TRF2^T188A^ cells transfected with either NT-siRNA or *cGAS*-siRNA. Data are means±SEM, *n* = 3–5 independent experiments, ns-non significant (*p* > 0.05), unpaired, two-tailed Student’s *t* test or Mann–Whitney *U* test.

To characterize the mechanisms linking telomere-induced DNA damage with the SASP in VSMCs, we examined the presence of cytosolic DNA, which can induce a pro-inflammatory phenotype via the cytoplasmic DNA sensor *cGAS-STING* pathway^16,18,22,24^. TRF2^T188A^ cells displayed cytoplasmic DNA fragments/micronuclei (MN), which stained positively for γ-H2AX and the heterochromatin marker H3K9me3 confirming their nuclear origin^22^ (Fig. 4a, Supplementary Figure 2a), with increased γ-H2AX^+^ and H3K9me3^+^ MN vs EV cells (Fig. 4b). Telo-FISH demonstrated that MN contained telomere sequences, and TRF2^T188A^ cells showed increased telomere^+^ MN compared to EV cells (Fig. 4c, Supplementary Figure 2b). TRF2^T188A^ VSMCs showed increased phosphorylation of TBK1 and its downstream mediator p65 NF-κB (Fig. 4d), suggesting *cGAS-STING-TBK1* pathway activation in these cells. Indeed, TRF2^T188A^ VSMCs showed increased mRNA expression of *IL1α*, *IL1β*, *IL8*, *CCL2*, and *CCL20* genes commonly associated with the SASP, but not the interferon-stimulated genes *interferon regulatory transcription factor 3* (*IRF*3), *IRF7*, or *Interferon-stimulated gene 56* (*ISG56*) (Fig. 4e). To examine whether the *cGAS-STING-TBK1* pathway is required for SASP stimulation in this system, we transiently silenced *cGAS* in EV and TRF2^T188A^ VSMCs; *cGAS* knockdown reduced TBK1 and p65 phosphorylation (Fig. 4f) and *IL1*α and *IL8* but not *CCL20* expression (Fig. 4g).

To confirm whether SIPS is also associated with cytoplasmic DNA and a SASP, we examined the presence of micronuclei and *cGAS-STING* pathway activation after doxorubicin treatment and recovery. Doxorubicin 1d + 21d recovery increased telomere^+^ MN but this was not observed after acute treatment (Supplementary Figure 3). Doxo-induced SIPS also induced *IL1α, IL1β, IL6, IL8, CCL2, CCL*20, and *CXCL10* (but not mRNAs for interferon-induced genes) despite no obvious changes in p60, p105, or p50 NF-κB isoforms (Supplementary Figure 3). In contrast, TBK1 phosphorylation was increased in SIPS compared to control cells, and *cGAS* silencing reduced both TBK1 phosphorylation and levels of *IL1α* and *IL8*, but not *CCL20* (Supplementary Figure 4). These data suggest that induction of the pro-inflammatory phenotype in two different models of hVSMC senescence is associated with cytoplasmic DNA and regulated in part by *cGAS*, but the SIPS-induced SASP is not associated with NF-κB activation.

### VSMC-specific TRF2^T188A^ promotes neointima formation with no change in clonality

We have previously shown that VSMC senescence promotes atherogenesis in mice^6^; however, it is unclear whether VSMC senescence mediates its effects through regulation of VSMC proliferation, induction of inflammation, or both, and whether VSMC senescence regulates clonality. Atherosclerosis models are associated with profound changes in lipid levels that drive inflammation, making interpretation of the independent effects of cell senescence on inflammation difficult. In contrast, injury models induce a neointima with normal lipid levels. We, therefore, examined the effect of VSMC senescence after ligation of the left common carotid artery (LCCA), a model that provokes rapid VSMC proliferation. *SM22α-TRF2*^*T188A*^ mice express human TRF2^T188A^ from a minimal *SM22α* promoter construct that is only expressed in large artery VSMCs^6^, whereas deletion of the CARG box means that expression is maintained after injury^30^. *SM22α-TRF2*^*T188A*^ were crossed with *Myh11-Cre*^*ERT2*^ *Rosa26*-Confetti multicolor reporter mice (Supplementary Figure 5a), to allow clonal tracing of VSMC progeny regardless of the continued expression of the Myh11 promoter^1^. Recombination at the reporter locus was induced by administration of tamoxifen between 6 and 8w of age and ligation performed 1w after the final tamoxifen injection. The LCCA and right common carotid artery (RCCA) of *SM22α-TRF2*^*T188A*^*/Myh11-Cre*^*ERTM*^ *Rosa26-Confetti (TRF2*^*T188A*^*)* and littermate controls were harvested 28 days after surgery. Recombination efficiency was similar in *TRF2*^*T188A*^ and control mice (78% vs 79%), with a similar expression frequency of each reporter protein between mouse genotypes (Supplementary Figure 5b–d).

**Fig. 5:**
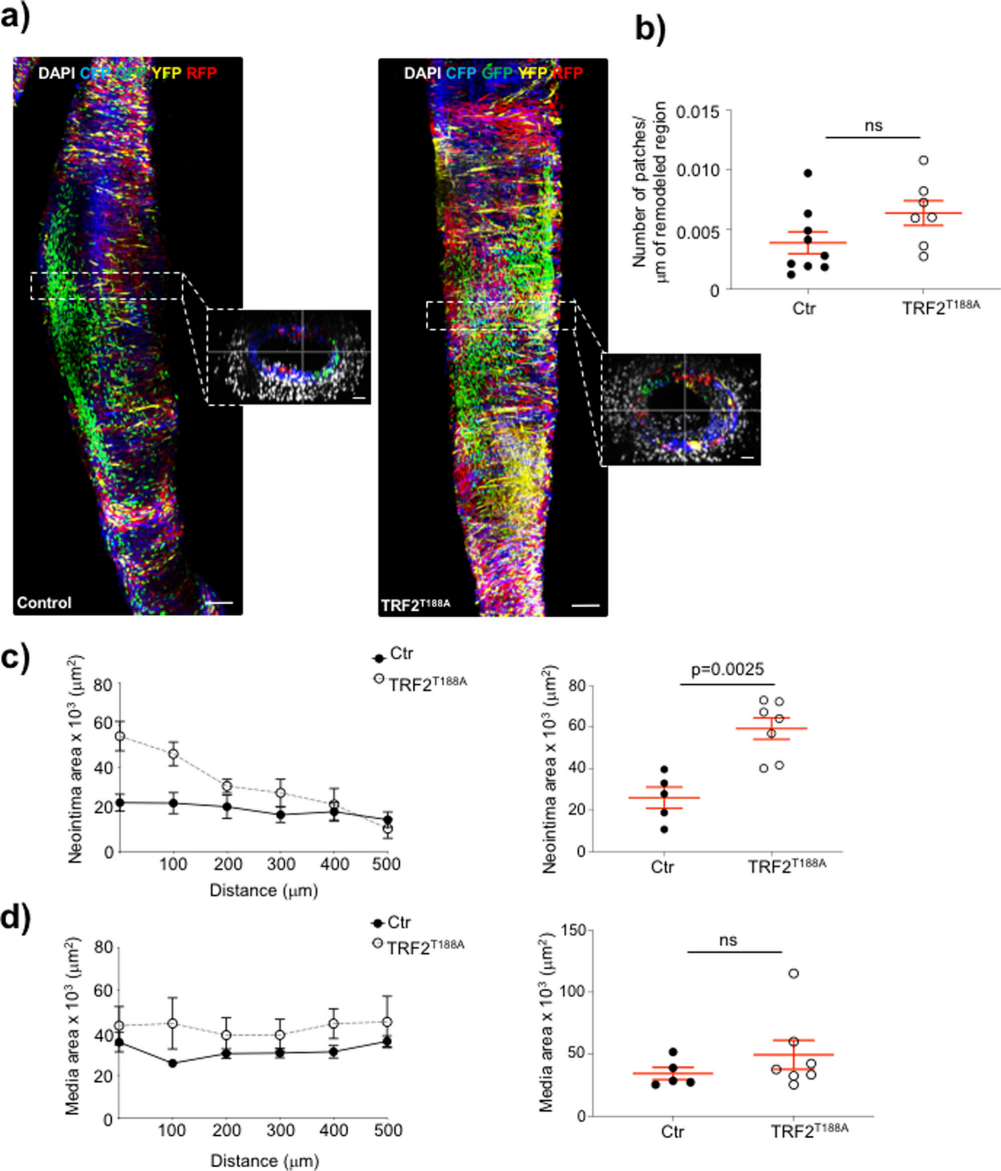
Telomere damage promotes neointima formation with no change in VSMC clonality. **a** Whole-mount images of the left common carotid artery (LCCA) of SM22α-TRF2^T188A^/Myh11-Cre^ERTM^ Rosa26-Confetti (TRF2^T188A^) mice and littermate controls (Ctr) 28d after carotid ligation. Images show maximum projection of the confocal Z-stack of the entire carotid artery and featuring CFP^+,^ YFP^+^, GFP^+^, RFP^+^ monochromatic patches. Outlined dotted regions represents cross-section. Scale bar = 100 μm and 30 μm (cross-section). **b** Number of monochromatic patches per μm of the remodeled region. **c** Mean neointimal area measured over six serial sections 100 μm apart or maximal neointimal area. **d** Mean medial area measured over six serial sections 100 μm apart or maximal medial area. Data shown are means ± SEM, *n* ≥ 5 mice per group, ns-non significant (*p* > 0.05), unpaired, two-tailed Student’s *t* test or Mann–Whitney *U* test.

Carotid ligation induces oligoclonal proliferation of VSMCs, resulting in monochromatic patches of VSMCs derived from a small number of pre-existing VSMCs^1^. Whole-mount confocal microscopic analysis of LCCAs showed that *TRF2*^*T188A*^ mice had similar numbers of monochromatic patches in the remodeled region compared with littermate controls (Fig. 5a, b). However, morphometric analysis of the remodeled area using six serial cryosections ~100 µm apart showed that *TRF2*^*T188A*^ mice had a significantly larger neointimal area than littermate controls (Fig. 5c). The medial area was similar between *TRF2*^*T188A*^ and control mice (Fig. 5d), but the external and internal elastic laminae (EEL and IEL) areas were increased in *TRF2*^*T188A*^ mice, with no difference in lumen area (Supplementary Figure 6). These data suggest that VSMC senescence promotes neointimal formation and outward remodeling in response to vascular injury, but does not change VSMC oligoclonality.

### VSMC-specific TRF2^T188A^ expression recruits inflammatory and immune cells to the vessel wall

**Fig. 6:**
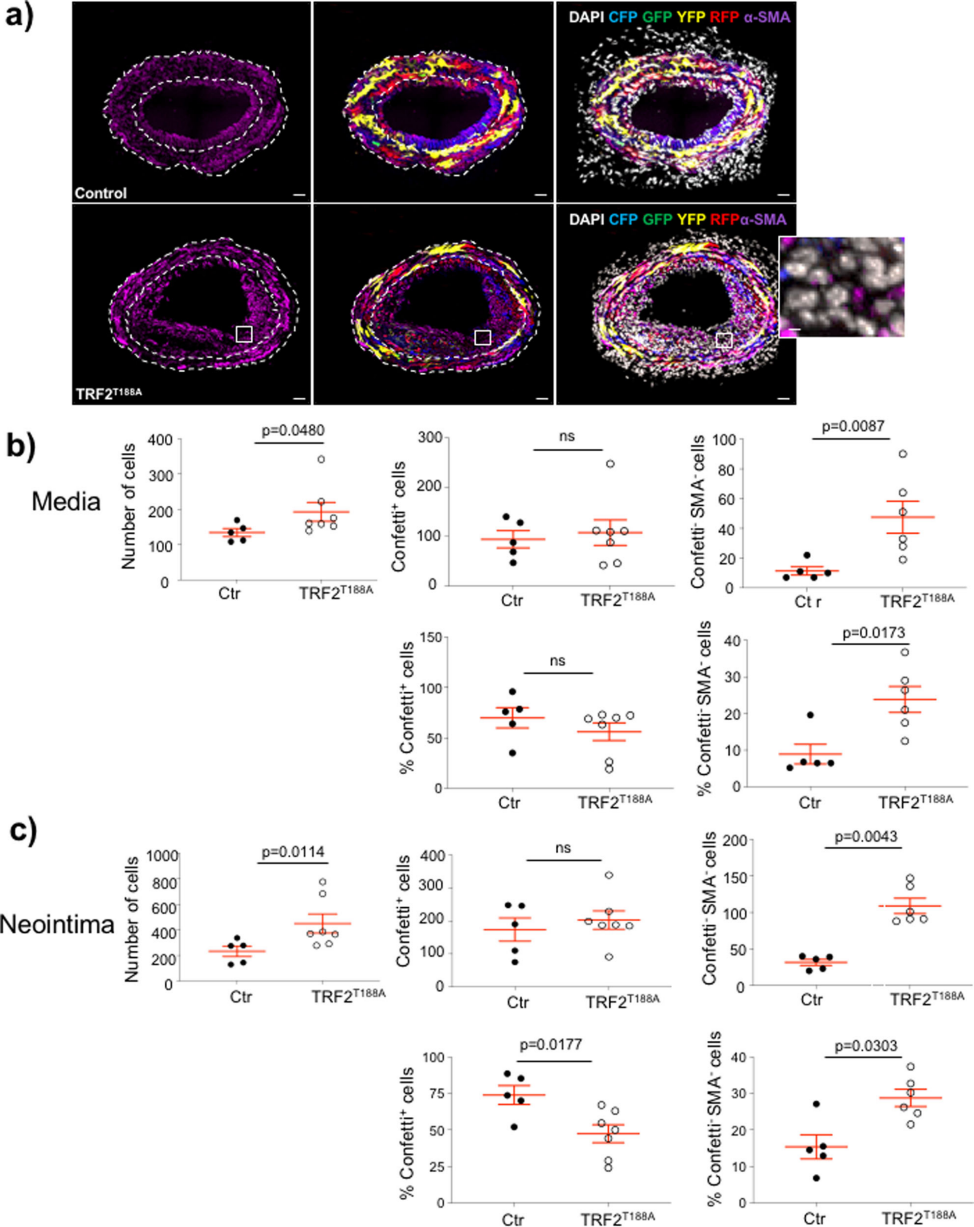
Telomere damage promotes accumulation of cells of non-VSMC origin in media and neointima. **a** Immunohistochemistry of LCCAs of TRF2^T188A^ and control mice 28d post ligation for confetti^+^ cells, α-SMA (magenta), and DAPI (white). Outlined regions show magnified confetti^−^/αSMA^−^ cells. Scale bar = 30 μm and 5.6 μm (inset). Overall number and % of cells (DAPI), confetti^+^ VSMCs, and confetti^−^/αSMA^−^ non-VSMCs in the **b** media or **c** neointima. Data represent means±SEM, *n* ≥ 5 mice per group, ns-non significant (*p* > 0.05), unpaired, two-tailed Student’s *t* test or Mann–Whitney *U* test.

**Fig. 7:**
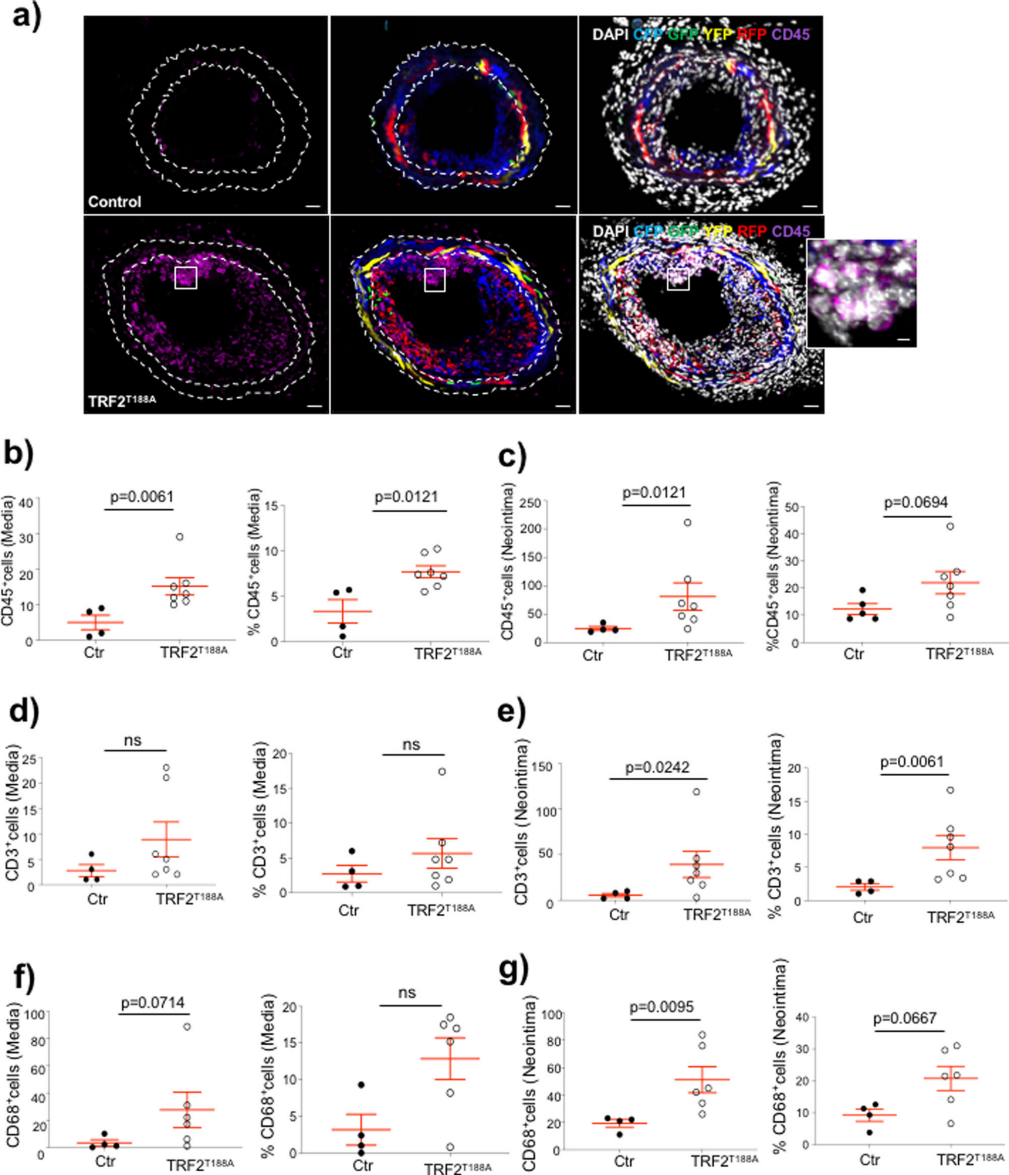
Increased inflammatory and immune cells in TRF2^T188A^ mice. **a** Immunohistochemistry of LCCAs of TRF2^T188A^ and control mice 28d post ligation stained with CD45 (magenta) and counterstained with DAPI (white). Outlined region shows magnified cluster of cells that are CD45^+^. Scale bar = 30 μm and 5.6 μm (inset). Total numbers and % of CD45^+^ cells in **b** media and **c** neointima, total numbers, and % of CD3^+^ cells in **d** media and **e** neointima or total numbers and % of CD68^+^ cells in **f** media and **g** neointima. Data represent means ± SEM, *n* ≥ 4 mice per group, unpaired, two-tailed Student’s *t* test or Mann–Whitney *U* test.

**Fig. 8:**
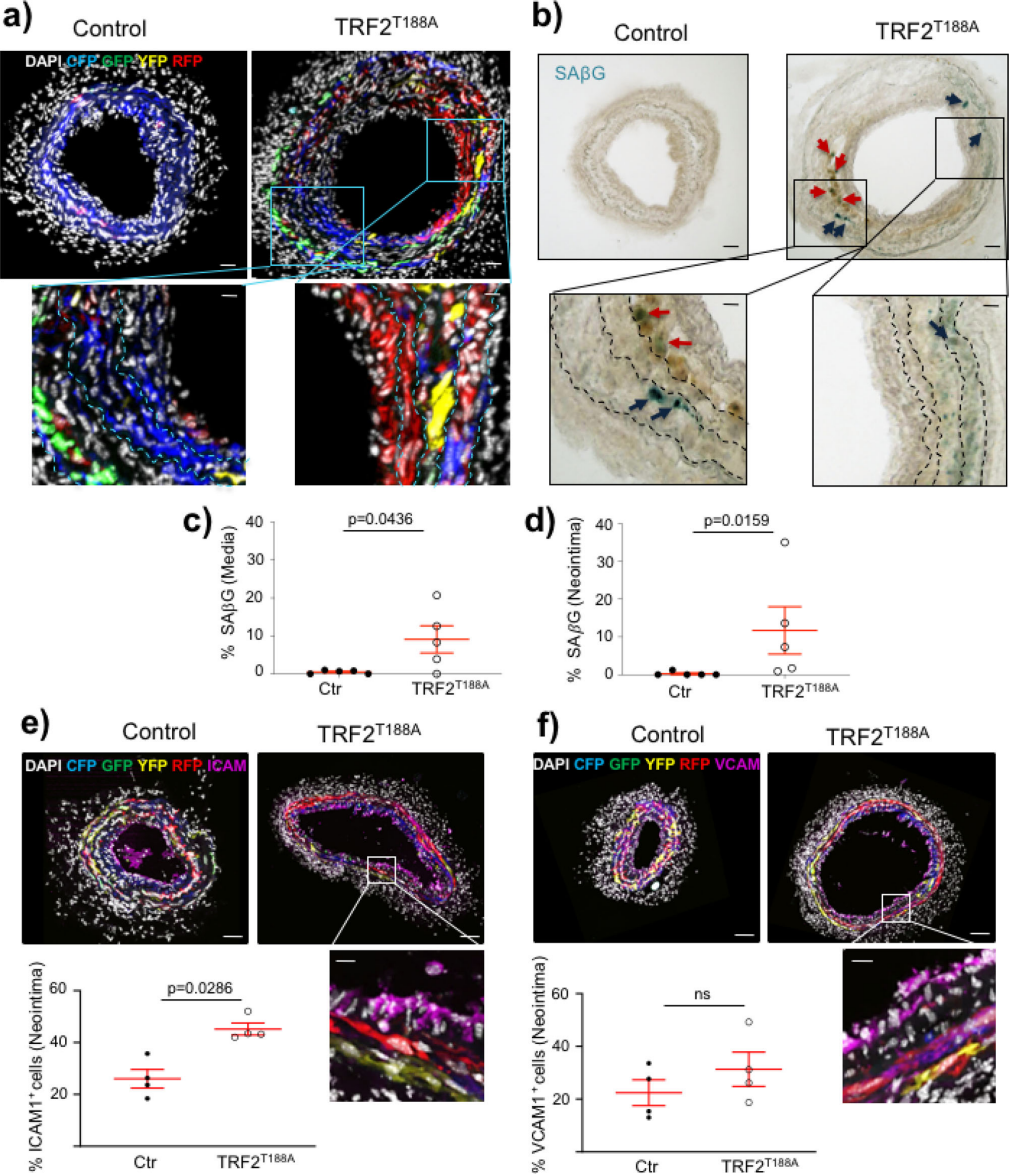
TRF2^T188A^ promotes senescence and expression of adhesion molecules in the neointima. **a** Cryosections of LCCAs, 28d post ligation in control and TRF2^T188A^ mice detected for confetti^+^ cells and DAPI (white). Scale bar = 30 μm and 13 μm (inset). **b** Cytochemical staining of the same section with chromogenic SAβG. Outlined regions show magnified SAβG^+^ cells in the media (indicated by blue arrows) or neointima (indicated by red arrows). Scale bar = 30 μm and 9.5 μm (inset). Dashed lines indicate elastic laminae. Quantification of SAβG^+^ cells in **c** media and **d** neointima, expressed as a percentage of SAβG^+^ cells over DAPI. **e**, **f** Immunohistochemistry for ICAM-1 (left) and VCAM-1 (right) (magenta) of LCCAs, 28d post ligation in control and TRF2^T188A^ mice, also detected for confetti^+^ cells. Scale bar=80 μm and 16 μm (inset). Quantification of ICAM-1^+^ or VCAM-1^+^ cells in neointima expressed as a percentage of ICAM-1^+^ or VCAM^+^ cells over DAPI. Data represent means±SEM, *n* = 4–5 mice per group, unpaired, two-tailed Student’s *t* test or Mann–Whitney *U* test.

We analyzed LCCA cryosections of TRF2^T188A^ and control mice at 28 days after ligation to assess whether the increased neointimal area is primarily owing to VSMCs, other cell types, or non-cellular material. The number of DAPI^+^ nuclei was increased in both media and neointima of *TRF2*^*T188A*^ mice; however, confetti^+^ VSMC numbers and % were similar in the media of *TRF2*^*T188A*^ and control mice and confetti^+^ VSMC % was reduced in the neointima of *TRF2*^*T188A*^ mice (Fig. 6a–c). To quantify non-VSMCs, we scored cells that were negative for both the confetti reporter and αSMA, as VSMCs might be misidentified due to <100% recombination of the confetti reporter. The number and % of non-VSMCs were significantly increased in both media and neointima of *TRF2*^*T188A*^ mice compared with controls (Fig. 6a–c), suggesting invasion by inflammatory/immune cells. Indeed, CD45^+^ cells were significantly increased in both media and neointima of *TRF2*^*T188A*^ mice (Fig. 7a–c), whereas CD3^+^ and CD68^+^ cells were significantly increased in neointimas of *TRF2*^*T188A*^ mice (Fig. 7d–g, Supplementary Figure 7). Cells expressing these leukocyte markers were negative for confetti, indicating that they were not derived from transdifferentiation of VSMCs. *TRF2*^*T188A*^ mice also showed increased % SAβG areas in both media and neointima compared to littermate controls, including in regions populated by confetti^+^ cells, suggestive of senescent VSMCs (Fig. 8a–d), and increased expression of the intercellular adhesion molecule-1 (ICAM-1) but not vascular cell adhesion protein-1 (VCAM-1) (Fig. 8e, f). Both proteins were predominantly expressed by confetti-negative cells in the neointima, particularly at the luminal surface.

## Discussion

Aberrant VSMC proliferation in response to injury has long been considered a major driver of atherogenesis and neointimal formation^3^, yet both vascular pathologies are associated with oligoclonal VSMC proliferation^1,2^ and the presence of senescent cells^4,31^. Senescent cells accumulate early after injury and application of exogenous senescent VSMCs to the adventitia promotes neointimal formation despite few cells appearing in the neointima, suggesting paracrine effects rather than direct migration/proliferation^31^. Although persistent oxidative DNA damage leads to VSMC senescence and inflammation^32^, it is not known whether inflammation is mediated through DNA damage preferentially at telomeres or to general genomic DNA, the mechanisms involved, and the consequences of VSMC senescence in vivo after injury. In addition, the contribution of VSMC senescence-induced inflammation compared with other effects of VSMC senescence is not clear.

We report multiple findings regarding telomere damage and inflammation in VSMC senescence, and its relevance in vascular remodeling. We show that: (a) SIPS of human VSMCs is associated with persistent telomere damage, whereas overall DNA damage is repaired; (b) TRF2^T188A^ expression induces VSMC senescence where DNA damage is predominantly in telomeres, associated with the generation of telomeric dilncRNA; (c) human VSMC senescence is associated with activation of multiple genes associated with the SASP and telomere damage/repair: (d) human VSMC senescence is associated with micronuclei, some of which contain telomeric DNA, and induction of multiple SASP cytokines, in part mediated through activation of the *cGAS-STING* cytoplasmic DNA-sensing pathway; (e) VSMC-specific expression of TRF2^T188A^ in vivo increases neointima formation and outward remodeling, and induces recruitment of inflammatory/immune cells into the neointima, with no change in VSMC clonality; (f) inflammation is a major component of the disease-promoting effects of VSMC senescence.

VSMCs manifest a wide range of DNA damage, activation of a DDR, and senescence in vascular disease^33^. Furthermore, persistent DSBs^34^, telomere dysfunction^6^, and oxidative DNA damage^32^ can all promote VSMC senescence. Here, we show that the DDR associated with sublethal DNA damage is mostly resolved prior to VSMC senescence; in contrast, SIPS is associated with persistent telomere damage, similar to senescent fibroblasts exposed to genotoxic and oxidative stress^11,13^. Repair of telomere damage has some unique features compared with other parts of the genome, in large part due to the presence of shelterin proteins. In particular, TRF2 and Protection of telomeres 1 (POT1) proteins can suppress NHEJ at telomeres to prevent chromosome end fusions and genomic instability^9,35^, and the persistent telomere DDR observed in VSMC senescence and in plaque VSMCs may be due to reduced levels of shelterin components to inhibit NHEJ. For example, TRF2 deletion or mutation typically causes uncapped and dysfunctional telomeres^9^. In addition, we found that TRF2 is reduced in SIPS, whereas TRF2^T188A^ overexpression results in DSBs restricted mainly to telomeres.

A persistent DDR promotes cell senescence and has a pivotal role in the activation of the SASP. Nuclear DNA in the cytosol can activate the *cGAS-STING* cytoplasmic DNA-sensing pathway and induce type-I interferon through *IRF3* and pro-inflammatory responses via *NF-κB* (reviewed in ref. ^26^). We show that the SASP can be induced by both doxorubicin-induced SIPS and TRF2^T188A^ expression, and is associated with cytosolic dsDNA positive for H3K9me3 and telomere sequences, suggesting that they contain condensed chromatin. This pro-inflammatory phenotype was associated with activation of TBK1 and the *cGAS-STIN*G pathway, as deletion of *cGAS* abrogated *IL1α* and *IL8* induction after SIPS and TRF2^T188A^ expression, and NF-κB phosphorylation was increased by TRF2^T188A^ expression but not SIPS. There was no upregulation of type-I interferon-regulated genes, consistent with previous reports showing that micronuclei can prioritize NF-κB signaling over *IRF3*, in which activation is regulated by p38 MAPK^22,36^. Interestingly, not all SASP factors were reduced upon *cGAS* silencing, and differed between SIPS and TRF2^T188A^ expression, suggesting different downstream mediators. Indeed, DNA damage in keratinocytes results in activation of the innate immune response independently of *cGAS*, an effect mediated by *STING* with predominant action on NF-κB^37^.

The pro-inflammatory phenotype of senescent cells may elicit multiple beneficial effects on the local environment, for example, by facilitating wound healing, cell proliferation, cell plasticity, and tissue remodeling^21,38,39^. However, SASP cytokines can also activate adjacent cells to recruit immune cells that might promote vascular disease^40^. We find that VSMC-specific TRF2^T188A^ expression generates a larger neointima after flow cessation associated with greater outward remodeling, preserving the lumen. TRF2^T188A^ mice had similar numbers of monochromatic VSMC patches in the remodeled region, but increased CD45^+^ leukocytes in both media and neointima, and increased neointimal CD68^+^ and CD3^+^ cells, and increased ICAM-1 expression. We speculate that enforced telomere damage does not change early events associated with oligoclonal proliferation seen after injury^1^, but induces premature VSMC senescence, which produces SASP cytokines that activate neighboring cells to proliferate or trigger immune cell recruitment. Notably, we found increased SAβG activity in both media and neointima of TRF2^T188A^ mice, including in regions occupied by Confetti^+^ cells but that had no increase in macrophages, suggesting that VSMC senescence occurs in both compartments after injury.

Importantly, our data help elucidate the contribution of cell senescence to vascular pathology. Our results are consistent with previous studies showing that cells expressing senescence markers are seen early after arterial injury, but are infrequent in a mature neointima unless senescence is promoted artificially or by aging^31,41,42^. VSMC senescence promotes vascular disease, particularly in atherosclerosis; however, it is unclear whether the effects are mediated by a failure of normal VSMC function (for example, to proliferate) or by increased inflammation. We show that medial repopulation by VSMCs and the number of VSMCs in the intima are not changed by senescence induced by TRF2^T188A^ expression. In contrast, increased neointimal formation is associated with a large increase in immune/inflammatory cells, suggesting that the pro-inflammatory effects of senescence may be more important than failure to replicate, at least after injury in mice. Our data also indicate that certain types of DNA damage may affect the relative importance of inflammation vs. reduced VSMC function. In particular, we show that SIPS and disrupting shelterin proteins induce persistent telomere damage and telomere DDR signaling, and enforced telomere damage in VSMCs is profoundly pro-inflammatory.

**Fig. 9:**
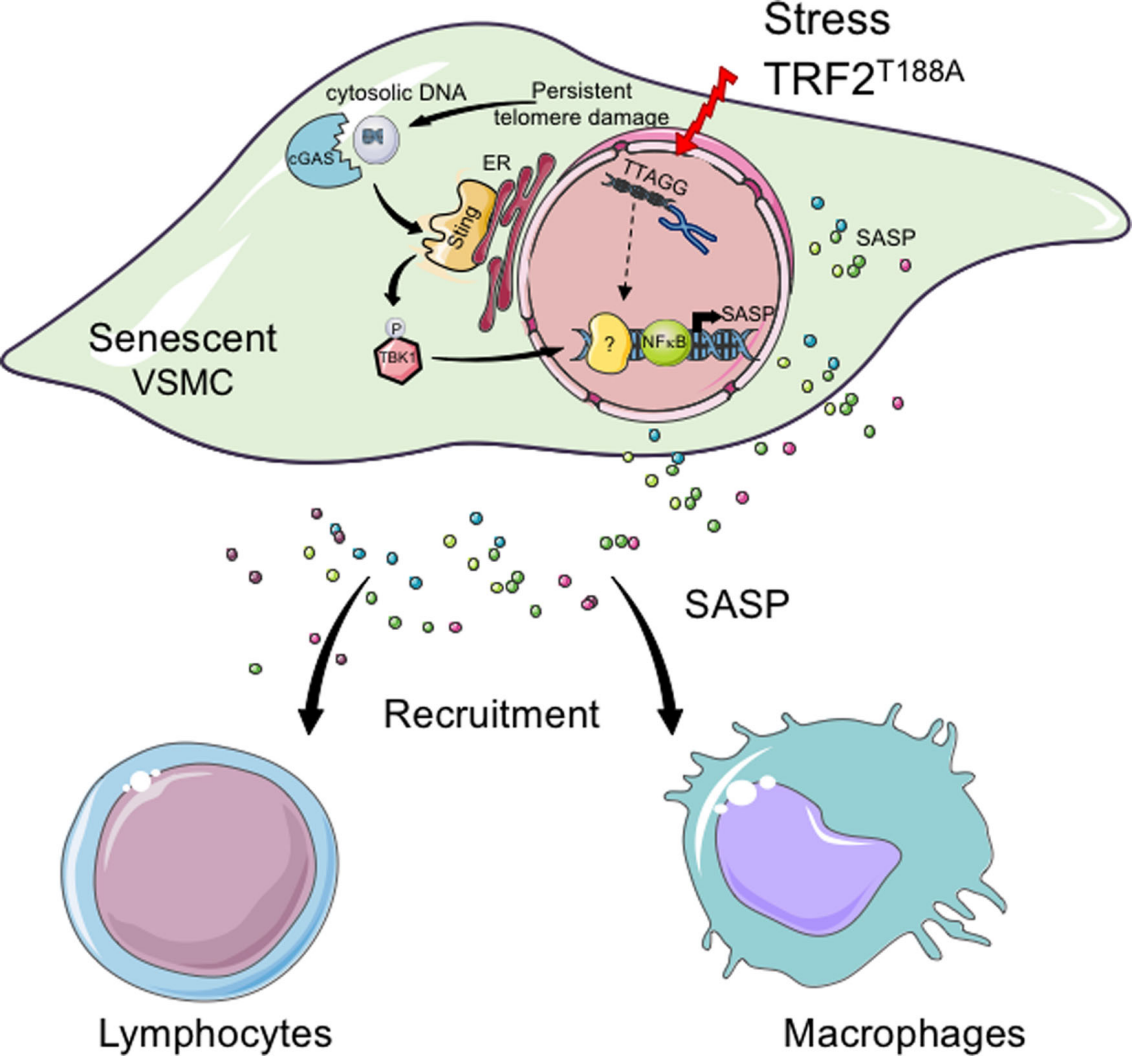
Model of telomere damage-induced VSMC senescence and inflammation. Stress-induced telomere damage or loss of TRF2 function promotes VSMC senescence and results in leakage of nuclear DNA to the cytosol. Cytoplasmic DNA fragments are then detected by the cGAS cytoplasmic DNA sensor pathway and activate phosphorylation of TBK1 and its downstream effectors, which in turn induce expression of SASP cytokines. Secreted SASP components mediate the recruitment of a variety of immune and inflammatory cells.

Our data support a general model where persistent telomere damage promotes VSMC senescence and results in the release of dsDNA (including of telomeric origin) to the cytosol; cytoplasmic DNA fragments then lead to activation of the *cGAS-STING-TBK1* pathway. The downstream signaling pathway then differs according to the stimulus or extent of DNA damage. TBK1 phosphorylation after TRF2^T188A^ expression leads to induction of *NF-κB* and downstream SASP cytokine genes, only some of which depend upon *cGAS*. Alternatively, telomere damage in SIPS activates downstream SASP cytokine genes, many of which depend upon *cGAS* but not *NF-κB*. Secreted SASP components can in turn recruit a variety of immune and inflammatory cells (Fig. 9).

In summary, we have identified that persistent telomere damage promotes VSMC senescence and inflammation. We propose that VSMC senescence exacerbates neointima formation after injury through the recruitment of macrophages and lymphocytes via SASP cytokines, which results in increased neointima formation and outward vascular remodeling. Our findings also help elucidate the mechanisms by which VSMC senescence promotes vascular disease.

## Methods

### Antibodies

For western blotting, antibodies were used as follows: TRF2 (1:500, Cell Signaling Technology, 13136), p16 (1:1000, Proteintech, 10883-1-AP), p21 Waf1/Cip1 (1:1000, Cell Signaling Technology, 2947), γ-H2AX (ser139) (1:500, Cell Signaling Technology, 9718), 53BP1 (1:1000, Novus, NB100-304), p53 (1:1000, Cell Signaling Technologies, 2524), P-p53 (1:1000, Cell Signaling Technologies, 9284), Myc (1:1000, Cell signaling, Technology 2276), P-TBK1 (Ser 172) (1:1000, Cell Signaling Technology, 5483), TBK1 (1:1000, Cell Signaling Technology, 3013), NF-κB P-p65 (Ser 536) (1:1000, Cell Signaling Technology, 3033), NF-κB p65 (1:1000, Cell Signaling Technology, 8242), NF-κB1 p105/p50 (1:1000, Cell Signaling Technology, 3035), cGAS (1:1000, Cell Signaling Technology, 15102), Actin (1:10 000, Sigma-Aldrich, A5-441), anti-rabbit HRP (1:3000, Cell Signaling Technology, 7074), anti-mouse HRP (1:3000, Cell Signaling Technology, 7076). For immunostaining: γ-H2AX (JBW301) (1:500, Merck, 05-636), H3K9me3 (1:400, Abcam, ab8898), 53BP1 (1:500, Novus, NB100-304), αSMA (Biotin) (1:500, Abcam, ab125057), CD45 (Alexa Fluor 647) (1:50, Biolegend, 103123), CD3 (Alexa Fluor 647) (1:50, Biolegend, 100209), CD68 (Alexa Fluor 647) (1:50, Biolegend, 137001), ICAM-1 (1:50, Biolegend, 116102), VCAM-1 (1:50, Biolegend, 105702).

### VSMC cultures

Human tissue was obtained under written informed consent using protocols approved by the Cambridge or Huntingdon Research Ethical Committee and conformed to the principles outlined in the Declaration of Helsinki. Primary human VSMC were isolated from aortas from patients undergoing cardiac transplant or aortic valve replacement, upon patient consent. After removing the endothelial layer and adventitia, tissues were cut into 2–3 mm² pieces, placed into a six-well plate containing 1.5 ml Smooth Muscle Cell Growth Medium 2 (Promocell) to allow cells to emerge. VSMCs derived from patients were tested for mycoplasm contamination, never pooled, and studied at passages 3–5. To perform SIPS experiments, human VSMCs were treated with either vehicle dimethylsulfoxide (Sigma-Aldrich) or doxorubicin (500 nM, Cayman Chemical) for 24 h, followed by 21-day recovery, with fresh medium replaced every 3 days. The replicative capacity of VSMCs was determined by measurement of CPD, which is defined as the total number of times the cells in a population have doubled^43^.

### Transfection, transduction, lentiviral production, and siRNA

Vectors were used as follows: pLenti-TRF2-Myc-DDK (OriGene), pMDLg/pRRE, pRSV-Rev, pMD2.G (Addgene). The pLenti-TRF2^T188A^-Myc-DDK was generated using PCR-based mutagenesis (QuickChange II SDM kit, Agilent), according to the manufacturer’s recommendations. The lentiviral vectors (3rd generation, pLenti-TRF2^T188A^-Myc-DDK or pLenti-Myc-DDK and pMDLg/pRRE, pRSV-Rev, pMD2.G) were packaged in HEK293FT cells (ATCC) using *Trans*IT-LT1 transfection Reagent (Mirus). Lentivirus-containing supernatant was harvested at 48 and 72 h following transfection. The supernatant was filtered through 0.45 µm pore size filter (Millipore) and concentrated down in Lenti-X Concentrator (Takara) according to the manufacturer’s recommendations. Human VSMC transduction was performed by mixing culture medium supplemented with 0.8 µg ml^−1^ polybrene with lentivirus (MOI 1–10). After 16 h lentiviral medium was replaced with fresh complete medium and cells were further selected with 1 µg ml^−1^ puromycin at 48 h post transduction. For transient silencing, human VSMCs were transfected with 50 nM of ON-TARGETplus SMARTPool *cGAS* or Non-targeted control siRNAs (Horizon) with 3 µl of Lipofectamine RNAiMAX (Thermo Fisher Scientific), according to the manufacturer’s instructions. Cells were maintained in culture for 48 h after transfection, before being analyzed.

### SAβG activity

SAβG activity was measured using Senescence Cells Histochemical Staining Kit (Sigma-Aldrich), according to the manufacturer’s recommendations and by flow cytometry using C_12_FDG^44^. For the SAβG staining, cells were fixed with 1× fixation buffer for 7 min at RT, washed 2× with phosphate-buffered saline (PBS) and incubated with the staining mixture (containing X-gal) at 37 °C (without CO_2_) for 5 h. Images were taken using a Nikon TMS-F microscope equipped with GXCAM LITE live camera and the percentage of SAβG-positive cells was quantified using ImageJ software. For flow cytometry, control and SIPS cells were pre-treated with either 100 nM of pH modulator-Bafilomycin A1 for 1 h followed by incubation with 33 µM of C_12_FDG for an additional 1 h before analysis on BD Accuri C6 flow cytometry system (BD biosciences). For the staining of the ligated carotid arteries, OCT-embedded pre-fixed tissue sections were washed 2× with PBS and incubated with the SAβG staining mixture in a humidified chamber overnight at 37 °C. The next day, sections were washed 2× with PBS, and images were taken using an Olympus BX51 microscope equipped with an Olympus Lumenera Infinity 3 digital camera. The same section was then imaged by confocal microscopy to identify VSMC-lineage traced cells in the SAβG-positive areas of the tissue.

### RNA isolation and RT-qPCR

Total RNA was isolated using miRNeasy kit (Qiagen), according to the manufacturer’s instructions and 0.5–1 µg of total RNA was converted to cDNA with Quantitect Reverse Transcription Kit (Qiagen). cDNA was diluted 1:10 before being amplified using Quantifast SYBR Green RT-PCR Kit (Qiagen) in a Corbett Life Science Rotor Gene 6000 PCR system (Qiagen). Relative gene expression was calculated using standard curves and the average of triplicate unknown samples normalized to the average of the housekeeping gene. Forward and reverse primers are listed in Supplementary Table 1. For strand-specific RT-qPCR, RNA samples were treated with DNase I (EN0525, Thermo Fisher Scientific) at 37 °C for 60 min to remove any residual genomic DNA contamination. Samples were then incubated with 1 ml 50 mM ethylenediaminetetraacetic acid for 10 min at 65 °C to inactivate the DNase. Next, 1 mg of RNA was reverse-transcribed using Superscript II Reverse transcriptase (18080044, Invitrogen) and RNaseOUT™ Recombinant Ribonuclease Inhibitor (10777019, Invitrogen) following the manufacturer’s protocol with strand-specific primers: RPP0 Rv for the detection of Rplp0 mRNA (control gene for normalization) and teloC Rv for the detection of G-rich telomeric transcripts. For the detection of C-rich telomeric transcripts, the teloG Rv primer was used. Next, 50 ng of cDNA per 20 ml reaction was used for real-time PCR using 2X LightCycler® 480 SYBR Green I Master (04707516001, Roche) and 10 mM telomere-specific (Telo Fw, Telo Rv) primers. RPP0 Fw and RPP0 Rv primers were used for the detection of Rplp0 transcripts for normalization (all primer sequences are listed in Supplementary Table 1). The cycling conditions for real-time PCR were as follows: preincubation at 95 °C for 5 min, 50 cycles of denaturation at 95 °C for 10 sec, annealing at 60 °C for 10 sec, and extension at 72 °C for 10 sec.

### Click-iT EdU incorporation assay

Cells were seeded onto coverslips and incubated for 24 h with 10 µM EdU, before being processed with Click-it EdU incorporation assay kit (Thermo Fisher Scientific), according to the manufacturer’s instructions.

### Western blotting

Cell lysates were prepared in radioimmunoprecipitation assay buffer, supplemented with proteinase inhibitors (1:100, Millipore) and phosphatase inhibitors (1:100, Millipore) and protein concentration determined using the (bicinchoninic acid assay BCA) method (23227, Pierce BCA protein assay kit, Thermo Fisher). Western blotting was performed using 4–12% polyacrylamide gels (or 3–8% tris-acetate gel for detection of 53BP1) with 10 µg of protein, followed by methanol-based wet transfer and chemiluminescence detection (Amersham ECL detection reagent, GE Healthcare). Uncropped gels are shown in Supplementary Figure 8.

### Immunofluorescence

Cells were grown on coverslips, fixed in 4% paraformaldehyde (PFA) for 10 min at room temperature (RT), washed in PBS, and permeablized with 0.3% Triton X-100 for 10 min. Next, cells were washed three times with PBS and then blocked in 3% bovine serum albumin (BSA) for 1 h at RT. Following blocking, cells were incubated with either primary or isotype-control antibodies in blocking buffer overnight at 4 °C. Cells were then washed with PBS, before being incubated with secondary antibodies for 1 h at RT. For nuclear staining, cells were washed with PBS, incubated for 10 min with 1 µg ml^−1^ of 4-6-diaminidino-2-phenylindole (DAPI) (Thermo Fisher Scientific), and mounted with ProLong Gold (Thermo Fisher Scientific). Slides were stored and imaged with confocal microscopy.

### Immuno-Telo-FISH

ImmunoFISH was performed with some changes to the protocol^45^. Human VSMCs were grown on coverslips and fixed and permeabilized with a 1:1 methanol to acetone solution. Subsequently, cells were washed, incubated with blocking buffer (2% Cold Water Fish Gelatin (Sigma) and 5% BSA in PBS), and stained with primary or isotype-control antibodies overnight at 4 °C. Cells were then washed and incubated with secondary antibody for 1 h at RT. All primary and secondary antibodies were diluted using a blocking buffer. Following secondary antibody incubation, cells were washed once with blocking buffer before being re-fixed with methanol/acetone for 2 min, treated with 0.1 mg ml^−1^ RNase solution (Invitrogen), and incubated for 20 min at 37 °C. After dehydration in ice-cold 70%, 85%, and 100% ethanol, coverslips were air-dried prior to hybridization with a Cy3-TelC PNA probe, following the manufacturer’s instructions (PNA Bio inc). Next, cells were then washed with PBS, incubated with DAPI, and mounted for imaging with confocal microscopy.

### Imaging and Image analysis

Slides were imaged with an SP8 confocal laser scanning microscope (Leica Microsystems) using a ×40 oil objective and optical section resolution of 1024 × 1024 pixels. Approximately 20 Z-stack images were acquired at 0.5 µm intervals between each stack. All images were processed using IMARIS 9.0.2 image analysis software (Bitplane). To quantify DNA damage foci, individual cells were identified with surface function using DAPI fluorescent signals, and a filter applied to remove incomplete cells imaged at the XY borders. To remove background and nonspecific signals outside of the nucleus, created surfaces were used to mask signals from the channel of interest. The threshold was adjusted for the masked channels to remove the background. Spots function was used to detect foci. Both surfaces and spots were then imported to Cells function where the number of foci was recorded. For telomere foci, colocalizing with DDR protein, both fluorescence signals (DNA damage protein and telomere signals) were subjected to Coloc function. The created colocalized channel was masked with created surfaces and processed as described above.

### Experimental animals

All animal experiments were performed under the Animals (Scientific Procedures) Act 1986 Amendment Regulations 2012 and were approved by Cambridge University Animal Welfare and Ethical Review Body (AWERB). *Myh-Cre*^*ERt2*^ animals are Y chromosome-linked; therefore all studies were performed using *SM22α-TRF2*^*T188A*^*/Myh-Cre*^*ERt2*^*/Rosa26*-Confetti^+^ males (heterozygous for *SM22α-TRF2*^*T188A*^ and *Rosa26*-Confetti^+^), which were generated by crossing *Myh-Cre*^*ERt2*^*/Rosa26*-Confetti^+^ males with *SM22α-TRF2*^*T188A*^ females, all on a C57Bl6 background. Recombination was induced by injecting 100 µL of 1 mg ml^−1^ tamoxifen (Sigma-Aldrich) intraperitoneally at 6–8 weeks of age for a total of 10 injections, followed by 1 week resting prior to carotid surgery. For carotid ligation, mice were given pre-operative buprenorphine (0.1 mg kg^−1^; subcutaneously) and anaesthetized with 2.5% inhalable isofluorane (maintained at 1.5%). The LCCA was exposed and ligated just below the bifurcation with a 6-0 silk suture. Following 28 days of recovery, both the RCCA and LCCA were collected and processed for confocal microscopy.

### Tissue processing

Dissected carotid arteries were fixed in 4% PFA in PBS for 20 min at RT, washed, and incubated with 1 µg ml^−1^ DAPI overnight at 4 °C. Following two washes with PBS, tissues were cleared overnight at 4 °C in RapiClear 1.52 (Sunjin lab) and whole-mounted for confocal microscopy. After image acquisition, carotid arteries were washed, cryo-protected overnight at 4 °C with 30% sucrose, and embedded in OCT compound (VWR), before being snap-frozen on dry ice or liquid nitrogen and stored at −80 °C.

### Immunostaining, microscopy, and image processing

Carotid arteries were cross-sectioned serially (14 µm thick) with the ligation site as the starting point. Sections were rinsed in PBS, permeablized (0.3% Triton X-100 in PBS for 20 min) and blocked for 1 h at RT in blocking buffer (1% BSA/10% normal goat serum (Dako) in PBS). Next, sections were incubated with either primary or isotype-control antibodies overnight at 4 °C, washed three times in PBS, and where needed incubated with secondary antibody for 1 h at RT. All primary and secondary antibodies were diluted using a blocking buffer. For nuclear staining, carotid arteries were incubated with DAPI (1 µg ml^−1^, 10 min at RT) and then mounted in RapiClear 1.52 for confocal imaging.

### Morphometric analysis

All morphometric analysis was performed using IMARIS 9.0.2 or Fiji (National Institute of Health). Six serial cryosections at 100 µm intervals were analyzed for neointimal, lumen, IEL, and EEL areas. The neointimal area was calculated by subtracting lumen from IEL circumference, and medial area by subtracting IEL from EEL circumference.

### RNAseq and bioinformatics

- RNA preparation
  mRNA was isolated using Nucleospin RNA columns (Macherey-Nagel) and concentrations determined by Nanodrop.
- Bulk RNAseq

RNA samples passed quality controls for RNA quality and integrity (Agilent Tapestation). RNA libraries were prepared using the TruSeq mRNA library preparation kit (Illumina, UK) and sequenced on a NextSeq sequencer (Illumina, UK) using a 75 cycle high output kit.

Post-sequencing, read quality was assessed using FastQC v.0.11.4^46^ and trimmed using Trim Galore v0.4.1^47^. Reads were mapped to the hGRCh38 genome using STAR v2.5.2a^48^. The number of reads that map to genes was calculated using HTSeq v0.6.0^49^. Normalization and differential gene expression analysis was performed using the R package edgeR, version 3.16.5 (R version 3.3.3). Genes with false discovery rate-adjusted *p* value<0.05 were identified as differentially expressed.

Hierarchical clustering and heatmap visualization of genes showing differential expression between SIPS (Dox 1 + 21d) and control samples were performed using the online tool Morpheus (https://software.broadinstitute.org/morpheus) (Broad Institute). Pathway analysis was performed separately for up and downregulated genes using the over-representation analysis tool in the Reactome Pathway Database v.73^50^ with Benjamini–Hochberg adjusted *p* value < 0.05. Selected pathways were visualized along the associated differentially expressed genes using CRAN R package GOplot^51^ v.1.0.2 in R v.4.0.2. For the downregulated genes, only differentially expressed genes associated with at least three of the selected pathways were shown in the GOplot.

### Statistics and reproducibility

Statistical analysis was calculated using GraphPad Prism 9.00 (GraphPad Software Inc.). Two group analysis was performed using unpaired two-tailed Student’s *t* test for normally distributed variables and Mann–Whitney test for data that were not normally distributed. Where three groups were compared, one-way ANOVA, with Dunnett’s correction was used, as detailed in figures. Experimental reproducibility was achieved by the following actions: (1) all in vitro data result from at least three independent experiments (defined as replicates) using human VSMCs derived from different donors, (2) the operator conducting the carotid ligation surgery was blinded to mouse genotype, and (3) the tissue sections were analyzed blindly. Animal randomization was achieved by the following measures: (1) wildtype littermates from *SM22α-TRF2*^*T188A*^*/Myh-Cre*^*ERt2*^*/Rosa26*-Confetti^+^ mice were used as controls, (2) both genotypes were housed in the same cages during colony expansion and experiments, and (3) underwent carotid ligation surgery and tissue collection on the same day. All data are shown in dotplots to demonstrate data distribution and represent individual data points, not technical replicates. Data are expressed as a mean ±SEM.

### Reporting summary

Further information on research design is available in the Nature Research Reporting Summary linked to this article.

## Supplementary information

Supplementary Information

Description of Additional Supplementary Files

Supplementary Data 1

Supplementary Data 2

Supplementary Data 3

Supplementary Data 4

Reporting Summary

## Data Availability

The RNAseq data set generated from human VSMCs undergoing SIPS or control cells has been deposited in the Gene Expression Omnibus (GEO) repository; accession number GSE171663. This data set and all other source data generated or analyzed during this study are included in this published article (and its supplementary information files Supplementary Data 1–4), but additional details can be obtained from the corresponding author on reasonable request.
