# Supplementary material for: Telomere damage promotes vascular smooth muscle cell senescence and immune cell recruitment after vessel injury
Source: Commun Biol. 2021 May 21;4:611. doi: 10.1038/s42003-021-02123-z (PMC8140103; doi:10.1038/s42003-021-02123-z)
Supplement: Supplementary file 1 — Supplementary Information [file 42003_2021_2123_MOESM1_ESM.pdf]

SUPPLEMENTARY INFORMATION

Telomere damage promotes vascular smooth muscle cell senescence and immune cell recruitment after vessel injury

Anna K Uryga[1#], Mandy OJ Grootaert[1#], Abel M Garrido[1], Sebnem Oc[1], Kirsty Foote[1], Joel Chappell[1], Alison Finigan[1], Francesca Rossiello[2], Fabrizio d'Adda di Fagagna[2,3], Dimitra Aravani[1], Helle F Jorgensen[1], Martin R Bennett[1]

[1]Division of Cardiovascular Medicine, Department of Medicine, University of Cambridge, Addenbrooke's Hospital, Cambridge, UK

[2]FIRC Institute of Molecular Oncology Foundation, IFOM Foundation, Milan, Italy
[3]Istituto di Genetica Molecolare, CNR-Consiglio Nazionale delle Ricerche, Pavia, 2700, Italy

Supplementary Figure 1

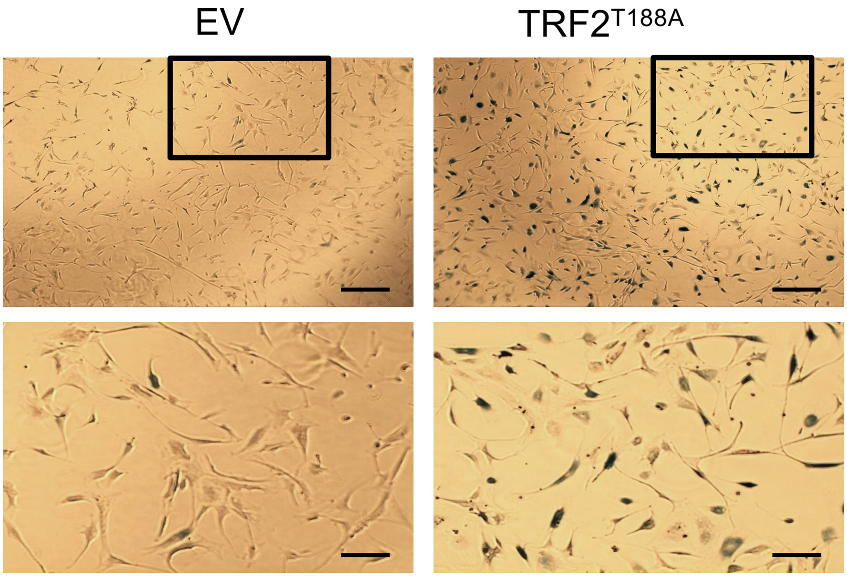

Supplementary Figure 1. SAβG activity in human VSMCs expressing an empty vector or TRF2^{T188A}
Human VSMCs infected with an empty vector (EV) or lentivirus expressing TRF2^{T188A}, and analysed for the senescence marker SAβG. Low-power (top, scale bar=150µm) and high-power (bottom, scale bar= 50µm) representative images of SAβG-positive cells.

Supplementary Figure 2

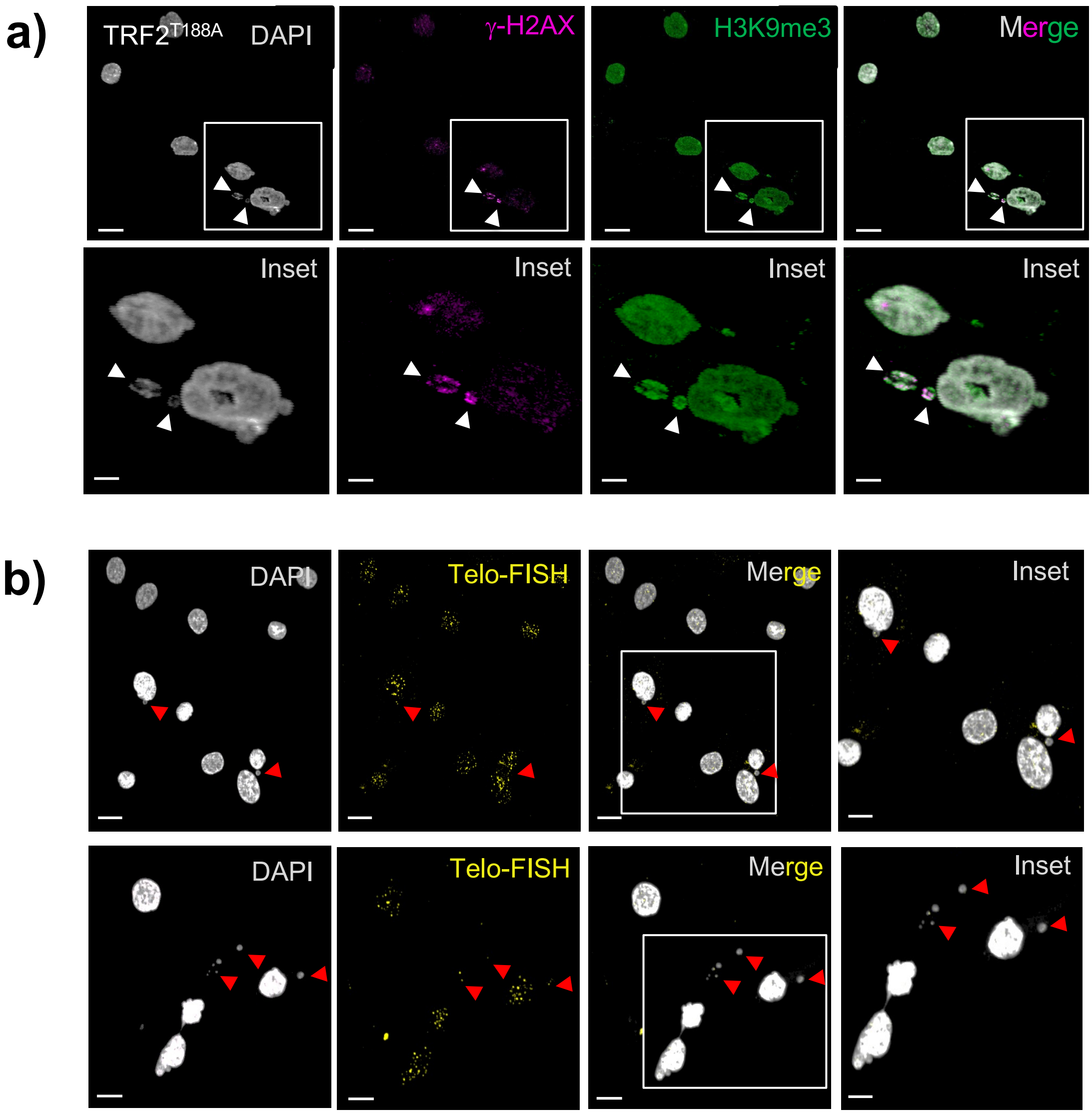

Supplementary Figure 2. Micronuclei in TRF2^{T188A} VSMCs

a) Examples of micronuclei (white arrowheads) seen at low power (upper panels) or high power view of inset image (lower panels) in TRF2^{T188A} VSMCs. Scale bars=20μm and 7.5μm (insets).

b) Examples of micronuclei containing telomere signals by TELO-FISH in TRF2^{T188A} VSMCs (red arrowheads) . High power view is shown on the right. Scale bars=20μm and 12.5μm (insets).

Supplementary Figure 3

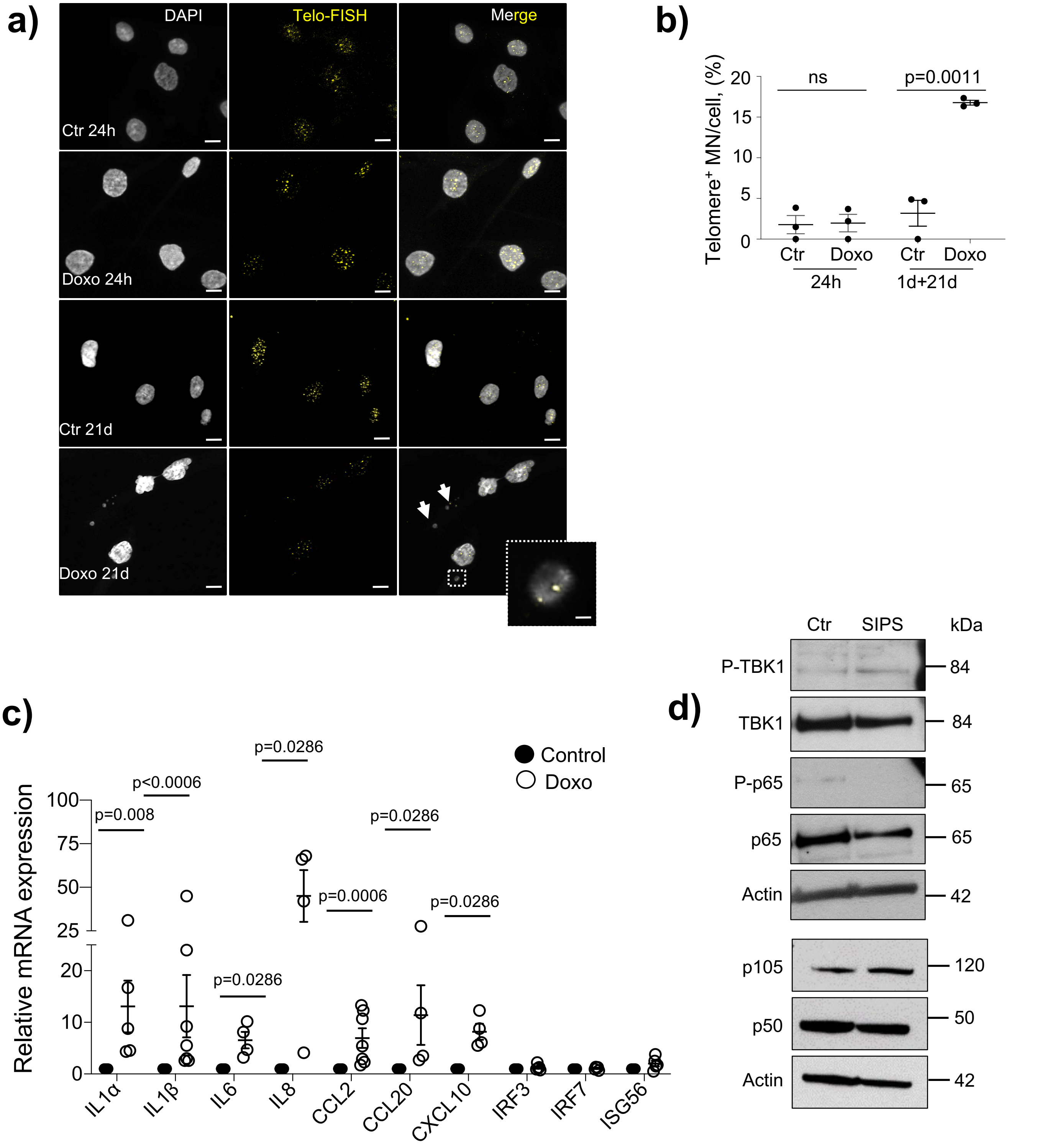

Supplementary Figure 3. Doxorubicin-induced premature senescence promotes SASP

a) Telo-FISH and **b)** quantification of Telomere+ micronuclei (MN)/cell in control cells or those undergoing doxo24h treatment or doxo-induced SIPs (Doxo1d+21d). DAPI (white) and telo-FISH (yellow). Arrows indicate MN. Scale bars=10μm and 1.25μm (inset). **c)** Relative mRNA expression of selected cytokines in control hVSMCs or those undergoing doxo-induced SIPS (Doxo1d+21d). n=4-7 independent experiments. **d)** Western blot for TBK1, p65 NF-κB and their phosphorylated forms and p50 and p105 NF-κB members in control hVSMCs or those undergoing doxo-induced SIPS. Data shown represent Means±SEM, n=3-5 independent experiments, ns-non significant ($p>0.05$), unpaired, two-tailed Student's t-test or Mann-Whitney U test.

Supplementary Figure 4

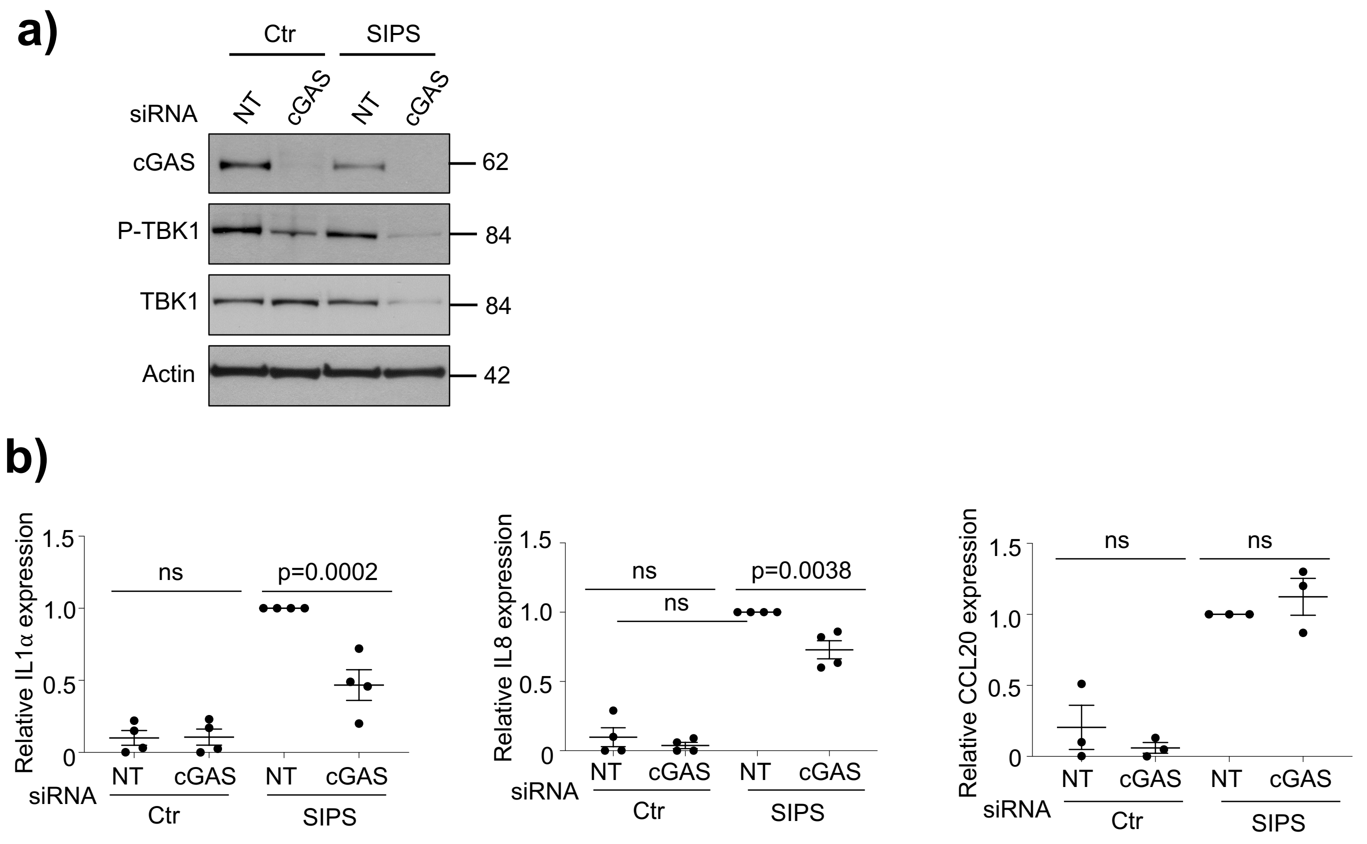

Supplementary Figure 4. Doxorubicin-induced premature senescence promotes SASP through cGAS-STING-TBK1 pathway

a) Western blot for cGAS, P-TBK1 and TBK1 in control hVSMCs or those undergoing doxo-induced SIPS transfected with non-targeting (NT)- or *cGAS*-siRNA. **b)** Relative mRNA expression of IL1α, IL8 and CCL20 in control hVSMCs or those undergoing doxo-induced SIPS transfected with non-targeting (NT)- or *cGAS-siRNA*. Data shown represent Means±SEM, n=3-5 independent experiments, ns-non significant (p>0.05), 1-way ANOVA with Bonferroni multiple corrections.

Supplementary Figure 5

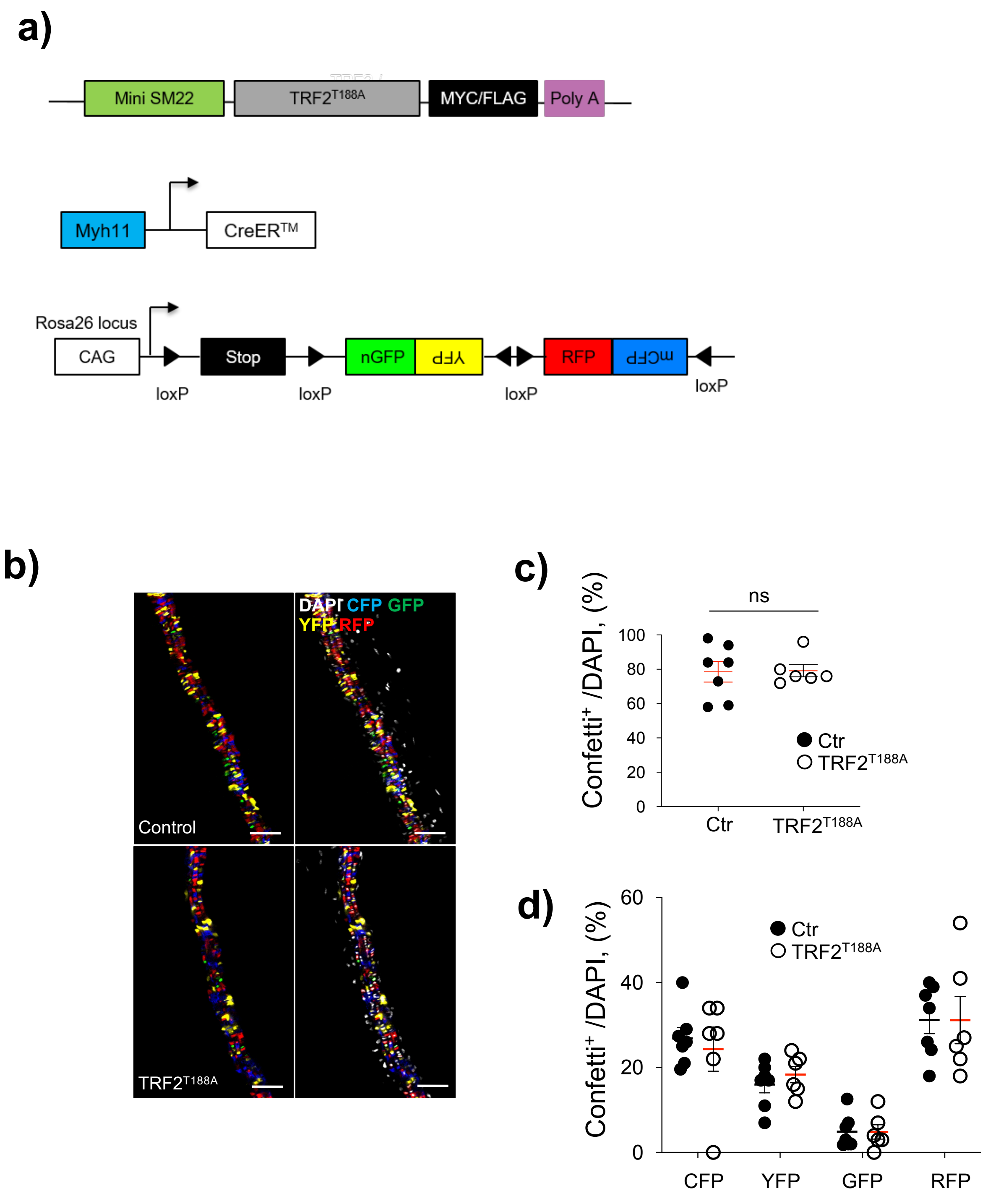

Supplementary Figure 5. VSMC labeling efficiency in TRF2^{T188A} mice and littermate controls.
a) Schematic of *SM22α-TRF2^{T188A}/Myh11-Cre^{ERTM}* and *Rosa26-Confetti* transgenic constructs. **b)** Right common carotid artery (RCCA) sections derived from *TRF2^{T188A}* mice and littermate controls. Scale bar=200μm. **c)** Percentage of total confetti^{+} cells and **d)** each confetti reporter color quantified on longitudinal sections of unligated RCCA of *TRF2^{T188A}* animals and littermate controls. Data shown in **(c-d)** represent Means±SEM, n≥6 mice for each group, ns-non significant ($p>0.05$), Mann-Whitney U test.

Supplementary Figure 6

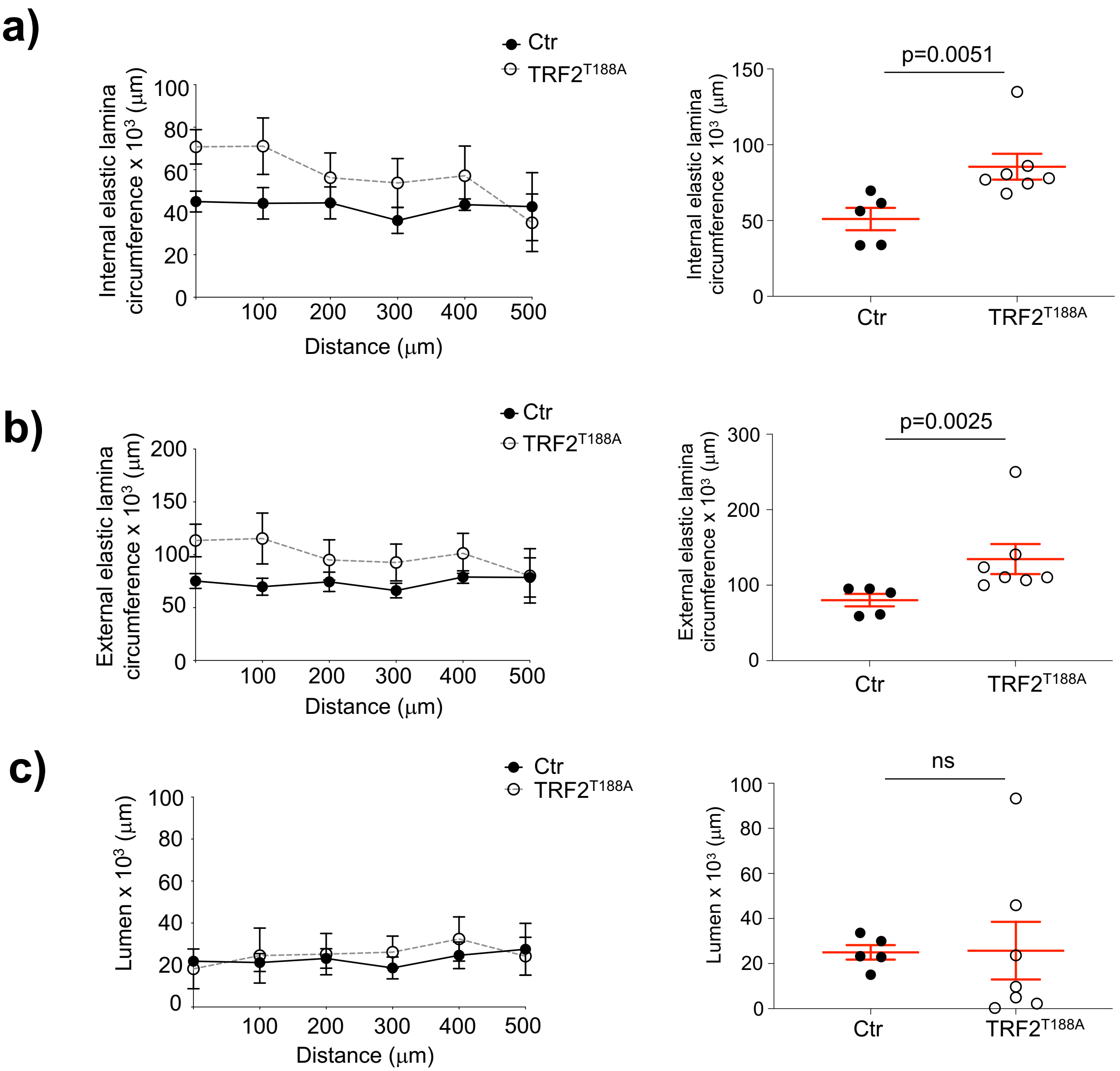

Supplementary Figure 6. TRF2^{T188A} promotes outward remodeling in response to injury
a) Internal elastic lamina, **b)** external elastic lamina and **c)** lumen area of Control (Ctr) or *TRF2^{T188A}* mice using six serial sections 100µm apart (left panels). Right panels represent maximum area. Data represent Means±SEM, n≥5 mice for group, ns-non significant (p>0.05), Mann-Whitney U test.

Supplementary Figure 7

a)

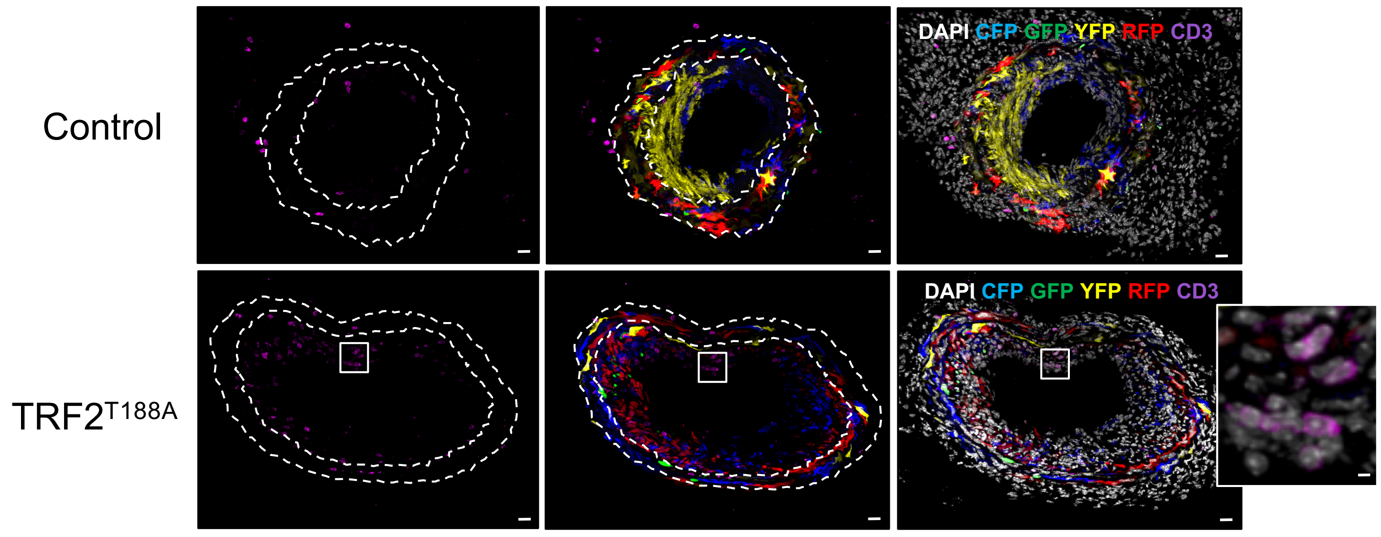

b)

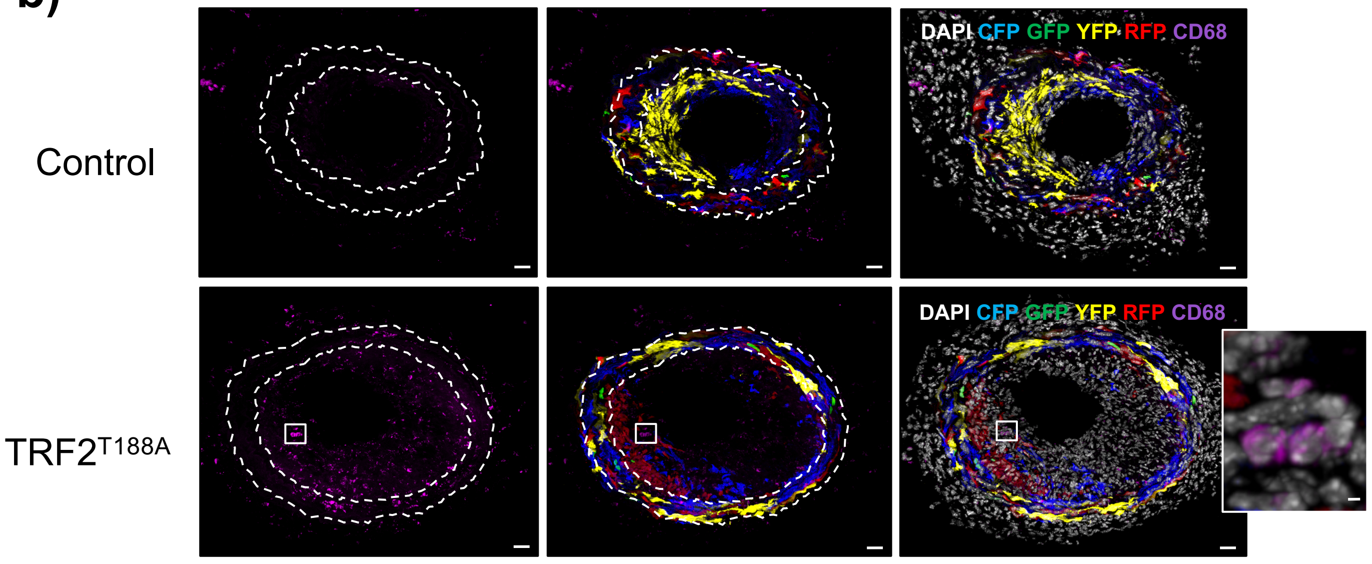

Supplementary Figure 7. Enhanced infiltration of lymphocytes and macrophages in TRF2^{T188A} mice.

LCCA cryosections of *TRF2^{T188A}* and control mice 28d following carotid ligation stained with **a)** CD3 (magenta) or **b)** CD68 (magenta) and counterstained with DAPI (white). Outlined regions show cluster of cells that are CD3^{+} or CD68^{+}. Scale bars=30μm and 4.8μm (inset).

Supplementary Figure 8

Uncropped gels

Fig 1g

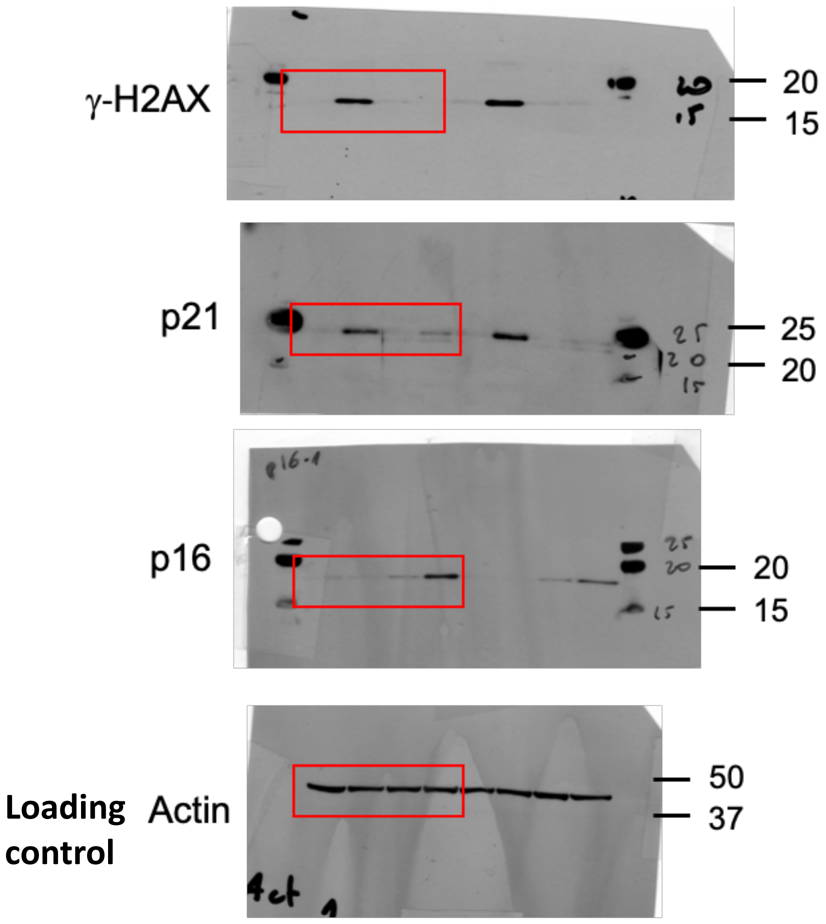
γ-H2AX
20
15
p21
25
20
p16
20
15
Loading control
Actin
50
37

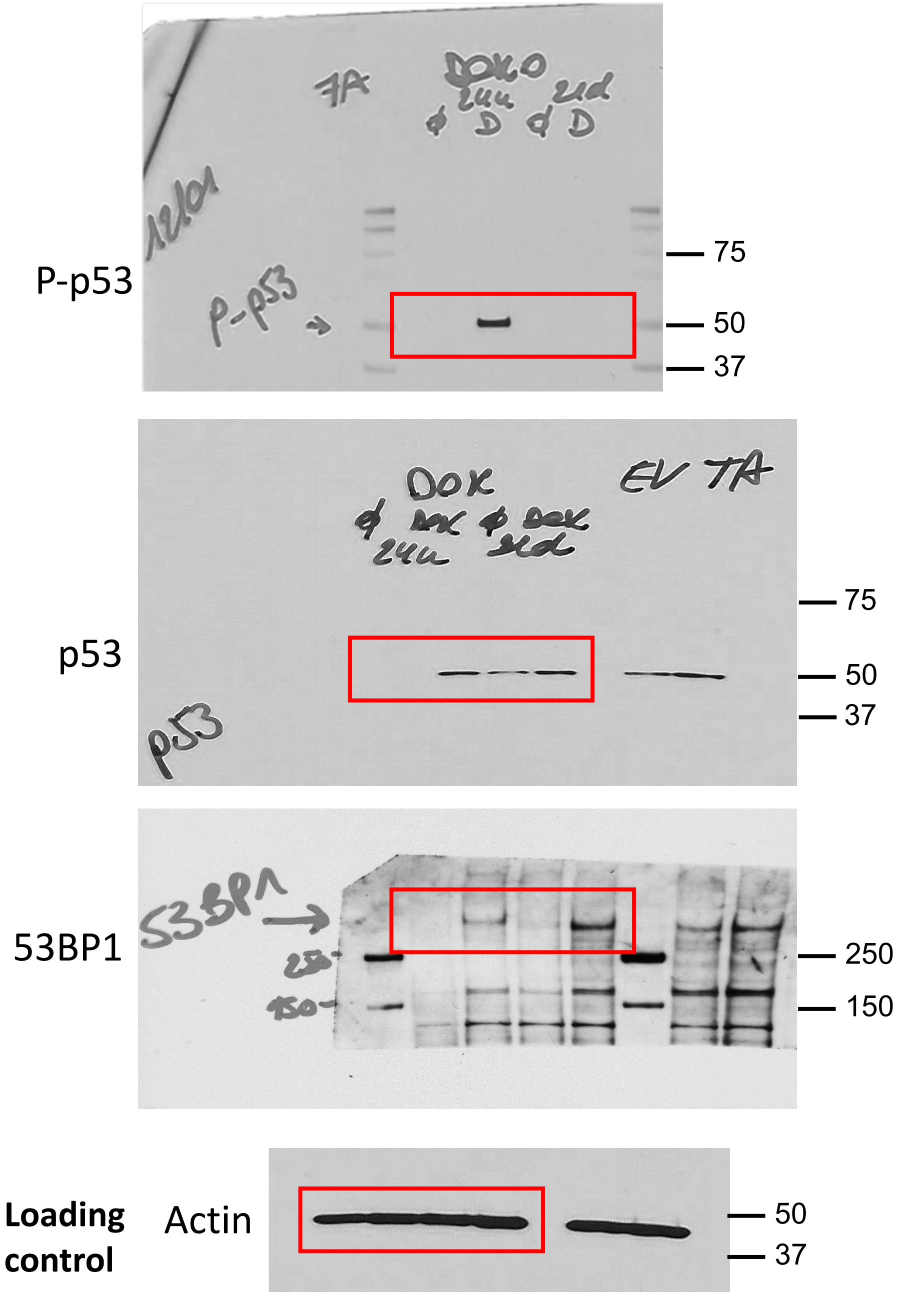
P-p53
75
50
37
p53
75
50
37
53BP1
250
150
Loading control
Actin
50
37

Fig 1j

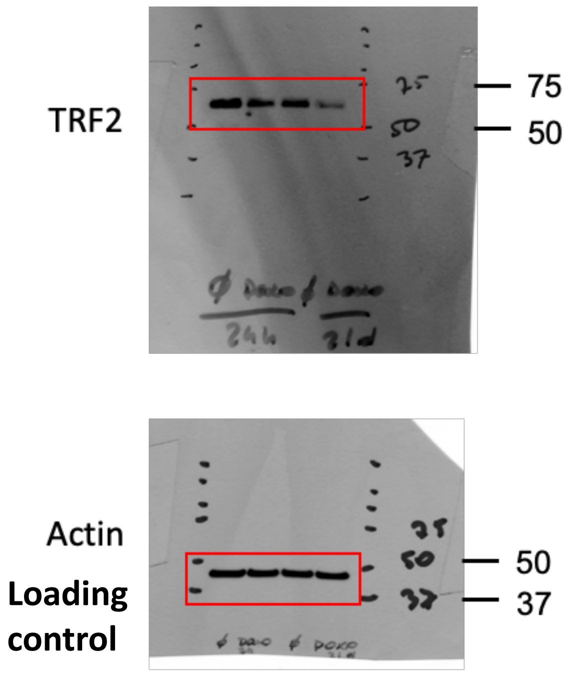
TRF2
75
50
Actin
Loading control
50
37

Fig 2d

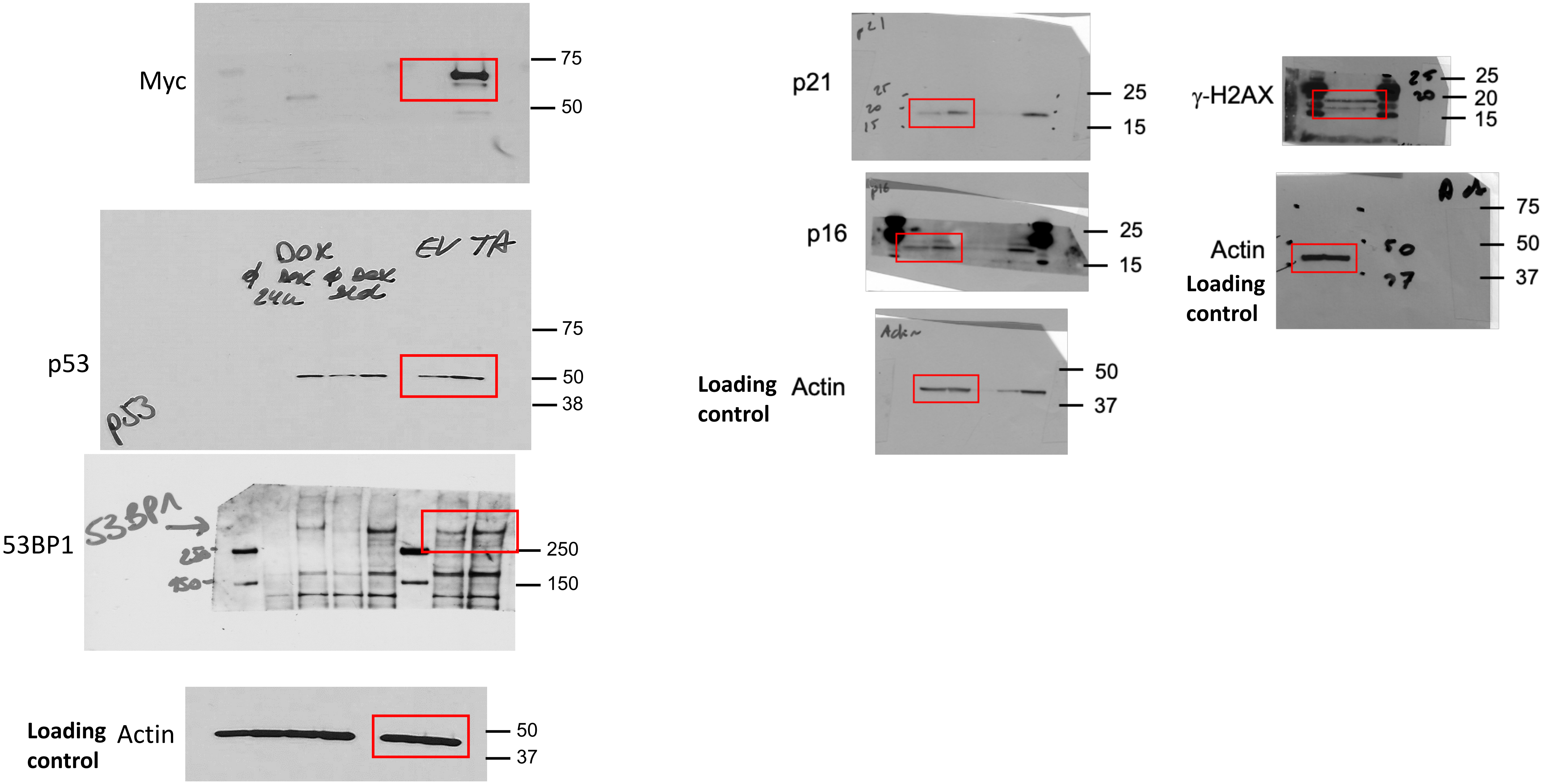

Myc
75
50
p53
75
50
38
53BP1
250
150
Loading control
Actin
50
37
p21
25
15
p16
25
15
Loading control
Actin
50
37
γ-H2AX
25
20
15
Actin
Loading control
75
50
37

Fig 4d

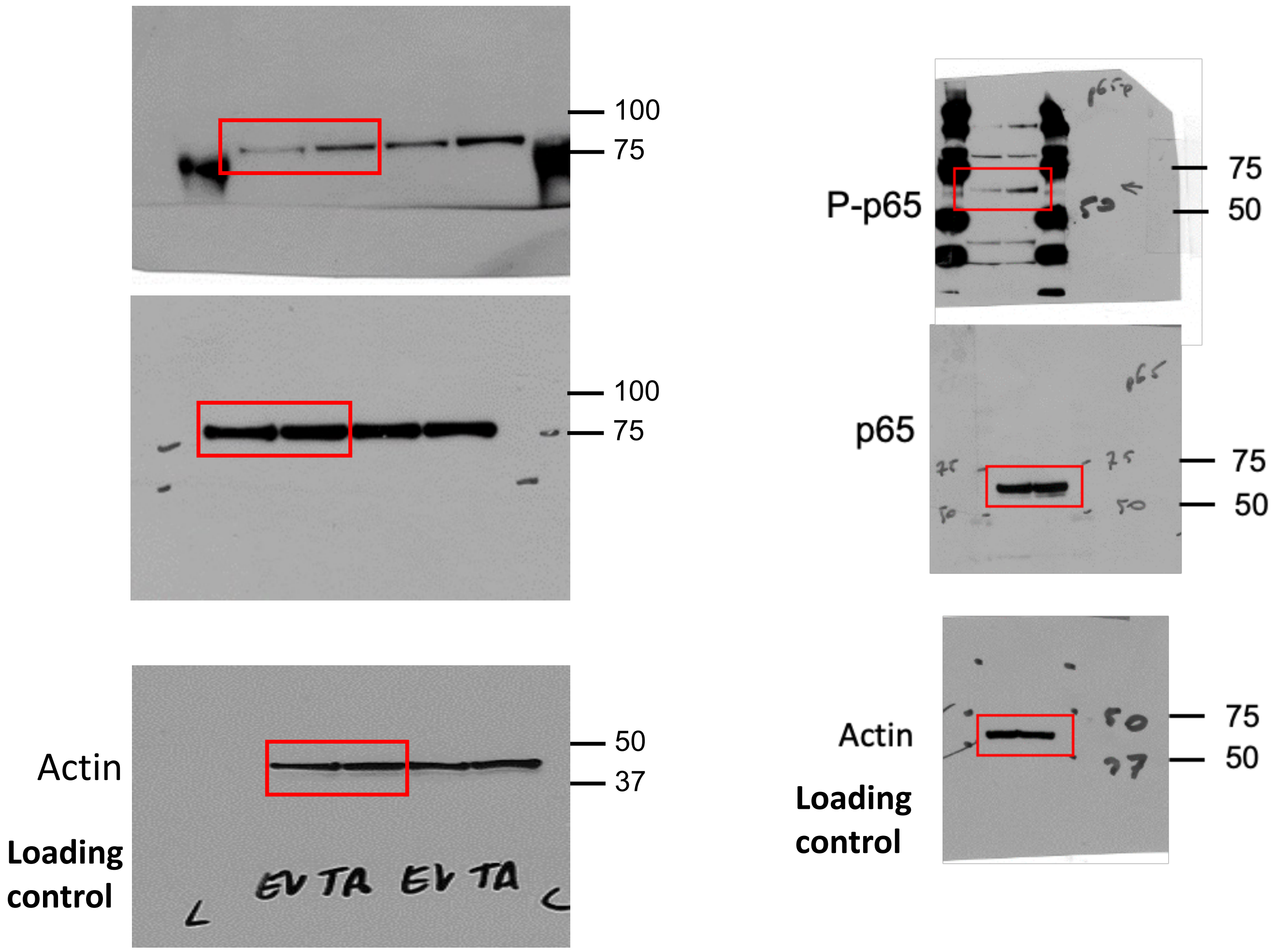
100
75
100
75
Actin
Loading control
50
37
EV TA EV TA
P-p65
75
50
p65
75
50
Actin
Loading control
75
50

Fig 4f

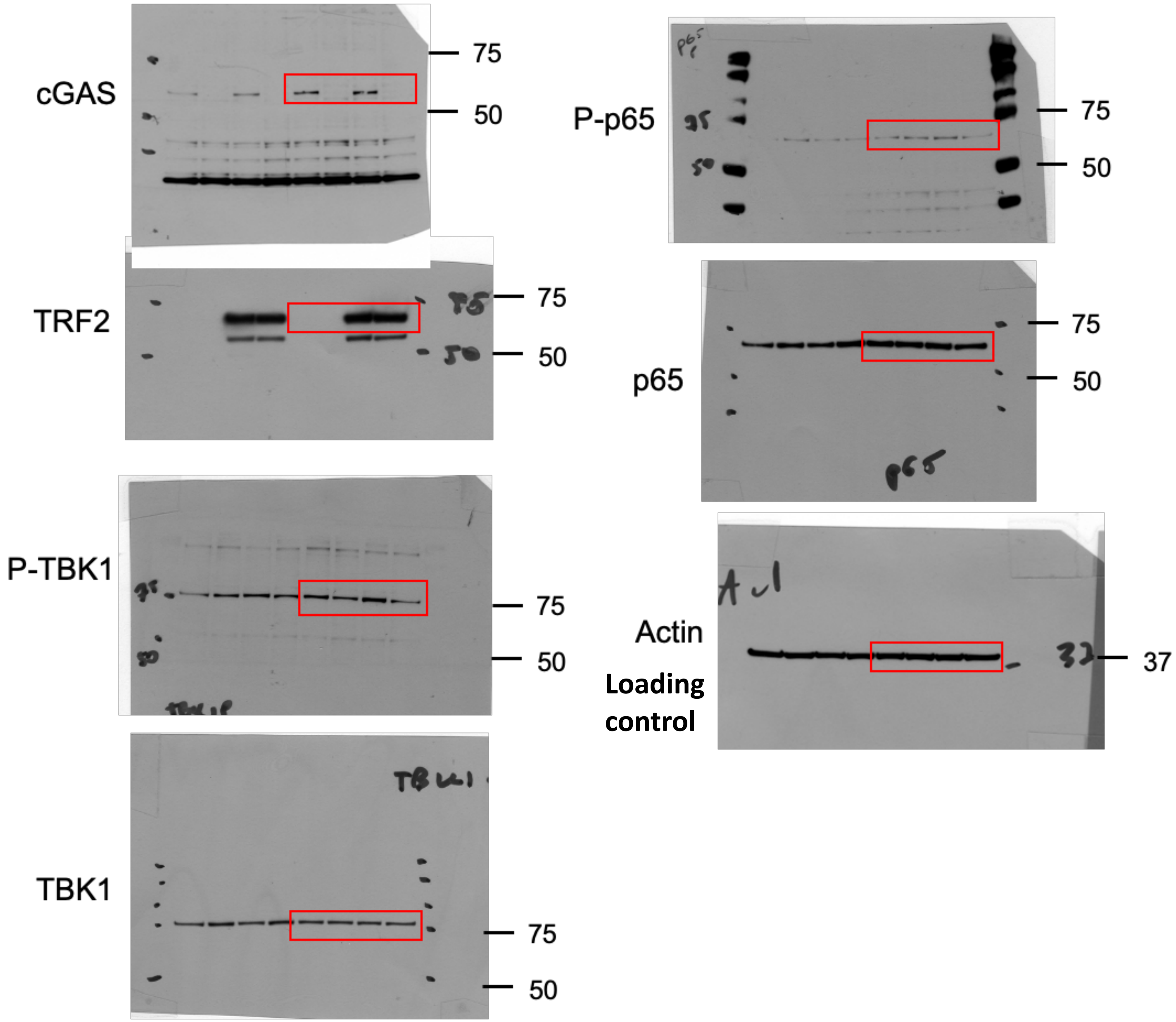

cGAS
75
50
TRF2
75
50
P-TBK1
75
50
TBK1
75
50
P-p65
75
50
p65
75
50
Actin
Loading control
37

Supplementary Fig 3d

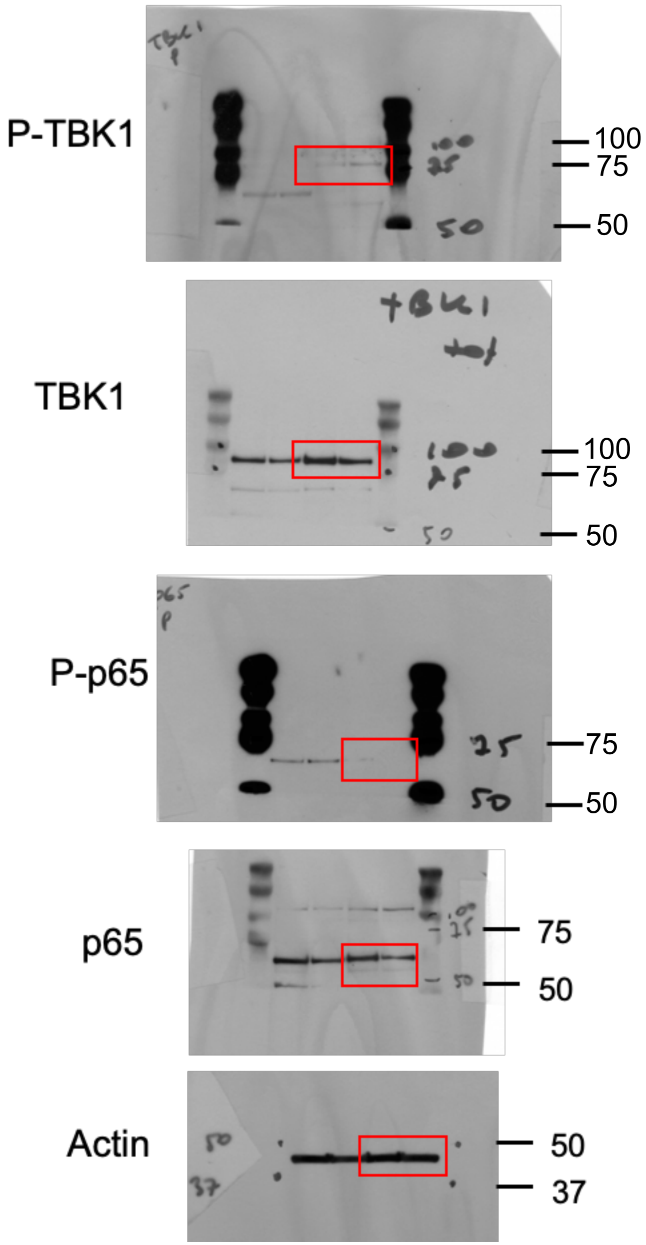

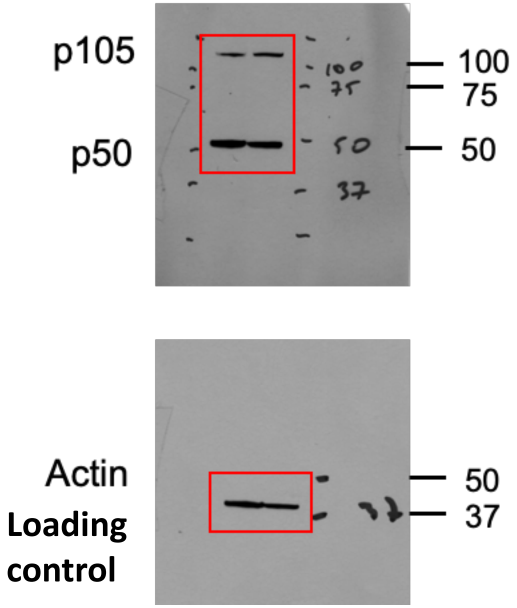

Supplementary Fig 4a

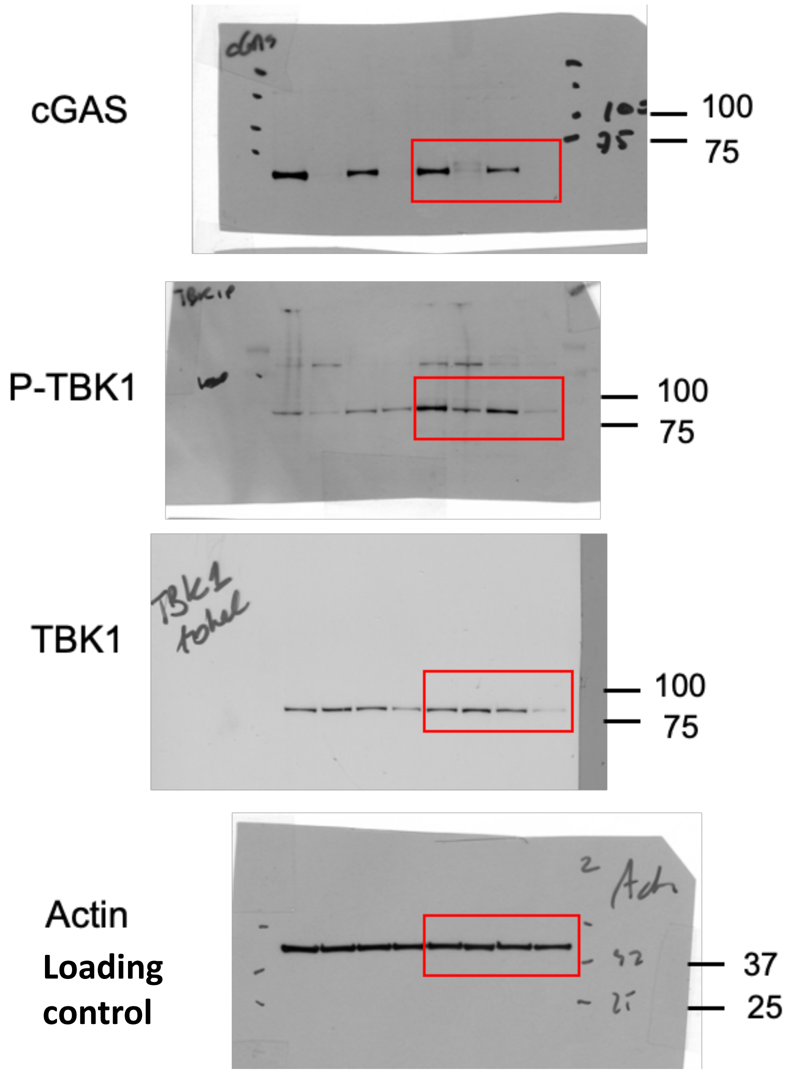

Human DNA primers	
IL1α	Fw 5'- ACTGCCCAAGATGAAGACCA -3'<br>Rv 5'- TGGTCTCACTACCTGTGATGG -3'
IL1β	Fw 5'- TCGCCAGTGAAATGATGGCT -3'<br>Rv 5'- TGGAAGGAGCACTTCATCTGTT -3'
IL8	Fw 5'- AGAGAGCTCTGTCTGGACCC -3'<br>Rv 5'- CTCAGCCCTCTTCAAAAACTTCT -3'
IL6	Fw 5'- CATCCTCGACGGCATCTCAG -3'<br>Rv 5'- TCACCAGGCAAGTCTCCTCA -3'
CCL2	Fw 5'- CTCAGCCAGATGCAATCAATG -3'<br>Rv 5'- CTTCTTTGGGACACTTGCTGC -3'
CCL20	Fw 5'- AACCATGTGCTGTACCAAGAGT -3'<br>Rv 5'- AAGTTGCTTGCTTCTGATTCGC -3'
CXCL10	Fw 5'- CCAGAATCGAAGGCCATCAA -3'<br>Rv 5'- CATTTCCTTGCTAACTGCTTTCAG -3'
IRF3	Fw 5'- TCGTGATGGTCAAGGTTGT-3'<br>Rv 5'- AGGTCCACAGTATTCTCCAG-3'
IRF7	Fw 5'- CCTCTCCAGATGCCAGTCCC -3'<br>Rv 5'- AAGGAGCCACTCTCCGAACA -3'
ISG56	Fw 5'- CAAAGGGCAAAACGAGGCAG -3'<br>Rv 5'- CCCAGGCATAGTTTCCCCAG -3'
p16	Fw 5'- CATAGATGCCGCGGAAGGT -3'<br>Rv 5'- AAGTTTCCCGAGGTTTCTCAGA -3'
p21	Fw 5'- GACTCTCAGGGTCGAAAACG -3'<br>Rv 5'- GGATTAGGGCTTCCTCTTGG -3'
RPL13A	Fw 5'- CGAGGTTGGCTGGAAGTACC -3'<br>Rv 5'- CCGTAGCCTCATGAGCTGTT -3'
TeloC (for RT)	Rv 5'- CCCTAACCCTAACCCTAA -3'
TeloG (for RT)	Rv 5'- TAGGGTTAGGGTTAGGG -3'
Telo (for qPCR)	Fw 5'- CGGTTTGTTTGGGTTTGGGTTTGCCTTTGCCTTTGGGTT -3'<br>Rv 5'- GGCTTGCCTTACCCTTACCCTTACCCTTACCCTTACCCT -3'
RPP0	Fw 5'- TTCATTGTGGGAGCAGAC -3'
RPP0	Rv 5'- CAGCAGTTTCTCCAGAGC -3'

Supplementary Table 1

List of QPCR primers
