# Supplementary material for: Telomere damage promotes vascular smooth muscle cell senescence and immune cell recruitment after vessel injury
Source: Commun Biol. 2021 May 21;4:611. doi: 10.1038/s42003-021-02123-z (PMC8140103; doi:10.1038/s42003-021-02123-z)
Supplement: Supplementary file 2 — Description of Additional Supplementary Files [file 42003_2021_2123_MOESM2_ESM.pdf]

Description of Additional Supplementary Files

File name: Supplementary data 1
Description: Hierarchical clustering and heatmap visualization of up- and down-regulated genes.

File name: Supplementary data 2
Description: Gene ontogeny (GO) term pathways for upregulated and down regulated genes.

File name: Supplementary data 3
Description: In vitro source data for Figures and Supplementary Figures.

File name: Supplementary data 4
Description: In vivo source data for Figures and Supplementary Figures.
