# Supplementary material for: Telomere damage promotes vascular smooth muscle cell senescence and immune cell recruitment after vessel injury
Source: Commun Biol. 2021 May 21;4:611. doi: 10.1038/s42003-021-02123-z (PMC8140103; doi:10.1038/s42003-021-02123-z)
Supplement: Supplementary file 7 — Reporting Summary [file 42003_2021_2123_MOESM7_ESM.pdf]

nature research

Corresponding author(s): Martin Bennett

Last updated by author(s): Apr 5, 2021

Reporting Summary

Nature Research wishes to improve the reproducibility of the work that we publish. This form provides structure for consistency and transparency in reporting. For further information on Nature Research policies, see our Editorial Policies and the Editorial Policy Checklist.

Statistics

For all statistical analyses, confirm that the following items are present in the figure legend, table legend, main text, or Methods section.

n/a	Confirmed	
☐	☒	The exact sample size (*n*) for each experimental group/condition, given as a discrete number and unit of measurement
☐	☒	A statement on whether measurements were taken from distinct samples or whether the same sample was measured repeatedly
☐	☒	The statistical test(s) used AND whether they are one- or two-sided<br>*Only common tests should be described solely by name; describe more complex techniques in the Methods section.*
☒	☐	A description of all covariates tested
☐	☒	A description of any assumptions or corrections, such as tests of normality and adjustment for multiple comparisons
☐	☒	A full description of the statistical parameters including central tendency (e.g. means) or other basic estimates (e.g. regression coefficient) AND variation (e.g. standard deviation) or associated estimates of uncertainty (e.g. confidence intervals)
☐	☒	For null hypothesis testing, the test statistic (e.g. *F*, *t*, *r*) with confidence intervals, effect sizes, degrees of freedom and *P* value noted<br>*Give P values as exact values whenever suitable.*
☒	☐	For Bayesian analysis, information on the choice of priors and Markov chain Monte Carlo settings
☒	☐	For hierarchical and complex designs, identification of the appropriate level for tests and full reporting of outcomes
☒	☐	Estimates of effect sizes (e.g. Cohen's *d*, Pearson's *r*), indicating how they were calculated

Our web collection on statistics for biologists contains articles on many of the points above.

Software and code

Policy information about availability of computer code

Data collection

Provide a description of all commercial, open source and custom code used to collect the data in this study, specifying the version used OR state that no software was used.

Data analysis

Provide a description of all commercial, open source and custom code used to analyse the data in this study, specifying the version used OR state that no software was used.

For manuscripts utilizing custom algorithms or software that are central to the research but not yet described in published literature, software must be made available to editors and reviewers. We strongly encourage code deposition in a community repository (e.g. GitHub). See the Nature Research guidelines for submitting code & software for further information.

Data

Policy information about availability of data

All manuscripts must include a data availability statement. This statement should provide the following information, where applicable:

- Accession codes, unique identifiers, or web links for publicly available datasets
- A list of figures that have associated raw data
- A description of any restrictions on data availability

The RNA-seq datasets generated from human VSMCs undergoing SIPS or control cells have been deposited in the Gene Expression Omnibus (GEO) repository; accession number is provided in the MS. Raw data files for in vitro and in vivo data are provided as Supplementary Data files.

Field-specific reporting

Please select the one below that is the best fit for your research. If you are not sure, read the appropriate sections before making your selection.

☒ Life sciences ☐ Behavioural & social sciences ☐ Ecological, evolutionary & environmental sciences

For a reference copy of the document with all sections, see nature.com/documents/nr-reporting-summary-flat.pdf

Life sciences study design

All studies must disclose on these points even when the disclosure is negative.

Sample size	We would attempt to demonstrate effects on total intimal or medial size between 2 experimental groups. Based on our previous studies and those of other groups, we expect a change of 100% in intimal size 4w after injury. If we use the equation for Student's t test for the difference of 2 means:-<br>x=1.96 - (m1-m2)/(2s2/n)<br>where m1= Mean of sample 1<br>m2=Mean of Sample 2<br>x=t statistic<br>s= Standard error of the means.<br>n=Sample size of both groups<br><br>To demonstrate a difference in the 2 means at p<0.05, t=1.65. The intimal size of a mouse carotid artery 4 weeks after injury is of the order of 0.1mm (s=0.006). To detect a 100% increase in this m2= 0.2mm. From the above equation, this gives us an n=5-6.
Data exclusions	We have excluded 8 mice that have gone through surgery, but did not show any vascular remodeling. This occurred in both control and T188A mice.
Replication	The number of experimental replicates is shown in the Figure legends
Randomization	Animals were housed in random order, WT and T188A were housed in the same cages. Experiments were performed within the same time frame and collected the same day.
Blinding	Blinding procedures with regard to masking of group assignment during outcome assessment were used

Reporting for specific materials, systems and methods

We require information from authors about some types of materials, experimental systems and methods used in many studies. Here, indicate whether each material, system or method listed is relevant to your study. If you are not sure if a list item applies to your research, read the appropriate section before selecting a response.

Materials & experimental systems

n/a	Involved in the study	
☐	☒	Antibodies
☐	☒	Eukaryotic cell lines
☒	☐	Palaeontology and archaeology
☐	☒	Animals and other organisms
☒	☐	Human research participants
☒	☐	Clinical data
☒	☐	Dual use research of concern

Methods

n/a	Involved in the study	
☒	☐	ChIP-seq
☒	☐	Flow cytometry
☒	☐	MRI-based neuroimaging

Antibodies

Antibodies used	For immunoblot<br>TRF2 (Cell Signaling Technology, Cat. number 13136, Clone D1Y5D) Lot:1<br>p16 (Proteintech, 10883-1-AP)<br>p21 Waf1/Cip1 (Cell Signaling Technology, Cat. number 2947, Clone 12D1) Lot:9<br>P-H2AX (ser139) (Cell Signaling Technology, Cat. number 9718, Clone 20E3) Lot:12<br>Myc (cell signalling, Cat. number 2276, Clone 9B11) Lot:6<br>P-TBK1 (Ser 172) (Cell Signaling Technology, Cat. number 5483, Clone D52C2) Lot:11<br>TBK1 (Cell Signaling Technology, Cat. number 3013) Lot:2<br>NF-kB P-p65 (Ser 536) (Cell Signaling Technology, Cat. number 3031, Clone 93H1) Lot:11<br>NF-kB p65 (Cell Signaling Technology, Cat. number 8242, Clone D14E12) Lot:13

NF-kB1 p105/p50 (Cell Signaling Technology, Cat. number 3035) Lot:5
cGAS (Cell Signaling Technology, Cat. number 15102, Clone D1D3G) Lot:3
53BP1 (Novus, Cat. number NB100-304) Lot:F-1
P-p53 (Cell Signaling Technologies, Cat. number 9284S) Lot:21
p53 (Cell Signaling Technologies, Cat. number 2524S) Lot:17
Actin (Sigma-Aldrich, Cat. number A5-441, Clone AC-15) Lot: 029M4883V
Anti-rabbit HRP (Cell Signaling Technology, Cat. number 7074) Lot:28
anti-mouse HRP (Cell Signaling Technology, Cat. number 7076) Lot:32
For immunofluorescence
P-H2AX (Merck, Cat. number 05-636, Clone JBW301) Lot: 3292608
H3K9me3 (Abcam, Cat. number ab8898)
53BP1 (Novus, Cat. number NB100-304) Lot: F-1
SMA (Biotin) (Abcam, Cat. number ab125057, Clone 1A4) Lot: GR316278-3
CD45 (Alexa Fluor 647) (Biolegend, Cat. number 103123) Lot: B283867
CD3 (Alexa Fluor 647) (Biolegend, Cat. number 100209) Lot: B290370
CD68 (Alexa Fluor 647) (Biolegend, Cat. number 137001, Clone FA11)
VCAM-1 (Biolegend, Cat. number 105702, Clone 429(MVCAM-A)) Lot: B217557
ICAM-1 (Biolegend, Cat. number 116102, Clone YN1/1.7.4) Lot: B243226

Validation

TRF2 - specificity for immunoblot validated in TRF2 knockdown (data not shown) and overexpressed human VSMC (Fig 4F)
p16 - used for immunoblot as a marker of senescence, Manufacturer validation "WB result of p16-INK4A antibody (10883-1-AP, 1:2,000) with si-Control and si-p16 transfected HEK- 293 cells", citation: S100A13 promotes senescence-associated secretory phenotype and cellular senescence via modulation of non-classical secretion of IL-1α. Su Y et al. Aging (Albany NY)
. 2019 Jan 23;11(2):549-572. doi: 10.18632/aging.101760

p21 Waf1/Cip1 -used for immunoblot as a marker of cell cycle arrest. Manufacturer validation "Western blot analysis from control HeLa cells (lane 1) or p21 Waf1/Cip1 knockout HeLa cells (lane 2) using p21 Waf1/Cip1 (12D1) Rabbit mAb (upper) or β-Actin (D6A8) Rabbit mAb #8457 (lower)", Cited: A phospho-switch controls RNF43-mediated degradation of Wnt receptors to suppress tumorigenesis. Tsukiyama T et al. Nat Commun. 2020 Sep 15;11(1):4586. doi: 10.1038/s41467-020-18257-3.PMID: 32934222

P-H2AX (ser139) - used for immunoblot as a marker of DNA damage. Manufacturer validation "Western blot analysis of extracts from untreated or UV-treated 293 cells, using Phospho-Histone H2A.X (Ser139) (20E3) Rabbit mAb (upper) or Histone H2A.X Antibody #2595 (lower)". Cited: Targeting codon 158 p53-mutant cancers via the induction of p53 acetylation. Kong LR et al. Nat Commun. 2020 Apr 29;11(1):2086. doi: 10.1038/s41467-020-15608-y. PMID: 32350249.

Myc- specificity for immunoblot validated in TRF2 overexpressed human VSMC (Fig 2D). Manufacturer validation "Western blot analysis of cell extracts expressing carboxy-terminal Myc-tagged protein (lane 1), amino-terminal Myc-tagged protein (lane 2) or control cell extracts (lane 3), using Myc-Tag (9B11) Mouse mAb" Cited: Histone deacetylase HDA-1 modulates mitochondrial stress response and longevity. Shao LW et al. Nat Commun. 2020 Sep 15;11(1):4639. doi: 10.1038/s41467-020-18501-w. PMID: 32934238.

P-TBK1- used for immunoblot. Manufacturer validaton "Western blot analysis of extracts from THP-1 cells differentiated with TPA #4174 (80 nM, overnight) followed by treatment with LPS (1 µg/ml), up to 24h, using Phospho-TBK1/NAK (Ser172) (D52C2) XP® Rabbit mAb (upper), or total TBK1/NAK (D1B4) Rabbit mAb #3504 (lower)" Cited: Pharmacological STING Activation Is a Potential Alternative to Overcome Drug-Resistance in Melanoma. Chipurupalli S et al. Front Oncol. 2020 May 14;10:758. doi: 10.3389/fonc.2020.00758. eCollection 2020. PMID: 32477956.

TBK1 - used for immunoblot. Manufacturer validation "Western blot analysis of extracts from HeLa (human), BaF3 (mouse) and KNRK (rat) cell lines, using TBK1/NAK Antibody" Cited Macrophages induce malignant traits in mammary epithelium via IKKε/TBK1 kinases and the serine biosynthesis pathway. Wilcz-Villega E et al. MBO Mol Med. 2020 Feb 7;12(2):e10491. doi: 10.15252/emmm.201910491. Epub 2020 Jan 13.PMID: 31930708.

NF-kB P-p65 (Ser 536)- used for immunoblot. Manufacturer validation "Western blot analysis of extracts from HeLa and NIH/3T3 cells, untreated or TNF-α treated (#2169, 20 ng/ml for 5 minutes), using Phospho-NF-κB p65 (Ser536) (93H1) Rabbit mAb (upper) or NF-κB p65 Antibody #3034 (lower)" Cited: Effects of the NF-κB/p53 signaling pathway on intervertebral disc nucleus pulposus degeneration. Zhang L et al. Mol Med Rep. 2020 Sep;22(3):1821-1830. doi: 10.3892/mmr.2020.11288. Epub 2020 Jun 30.

NF-kB p65 - used for immunoblot. Manufacturer validation "Western blot analysis of extracts from various cell lines using NF-κB p65 (D14E12) XP® Rabbit mAb". Cited: Effects of the NF-κB/p53 signaling pathway on intervertebral disc nucleus pulposus degeneration. Zhang L et al. Mol Med Rep. 2020 Sep;22(3):1821-1830. doi: 10.3892/mmr.2020.11288. Epub 2020 Jun 30.

NF-kB1 p105/p50 - used for immunoblot. Manufacturer validation "Western blot analysis of extracts from Vero cells, untreated or treated with TNF-α #2169 (20 ng/ml) for the times indicated, using Phospho-NF-κB p105 (Ser933) (18E6) Rabbit mAb #4806 (upper) and NF-κB p105/p50 Antibody #3035 (lower)" Cited: Molecular bases for HOIPINs-mediated inhibition of LUBAC and innate immune responses. Oikawa D et al. Commun Biol. 2020 Apr 3;3(1):163. doi: 10.1038/s42003-020-0882-8. PMID: 32246052

cGAS- specificity for immunoblot validated in cGAS knockdown (Fig 4F). Manufacturer validation "Western blot analysis of extracts from various cell lines using cGAS (D1D3G) Rabbit mAb" Cited: Small-molecule inhibition of aging-associated chromosomal instability delays cellular senescence. Barroso-Vilares M et al. EMBO Rep. 2020 May 6;21(5):e49248. doi: 10.15252/embr.201949248. Epub 2020 Mar 5. PMID: 32134180.

p53 and P-p53 – used for immunoblot. Validated in p53-/- mouse VSMCs (data not shown, Bennett et al, Vascular Smooth Muscle

Cell Senescence Promotes Atherosclerosis and Features of Plaque Vulnerability, Circulation, PMID: 26416809). Manufacturer validation "Western blot analysis of a p53 fusion protein, untreated or phosphorylated by DNA-PK, using Phospho-p53 (Ser15) Antibody (upper) and p53 Antibody #9282 (lower)." Cited: DNA damage-induced activation of p53 by the checkpoint kinase Chk2, Hirao et al, 2000, PMID: 10710310.

Actin- used for immunoblot. Manufacturer validation "Whole cell extract of human fibroblasts was separated on SDS-PAGE and blotted with Monoclonal Anti-β-Actin (Product No. A 5441). The antibody was developed with Goat anti-Mouse IgG, Peroxidase conjugate (Product No. A 9917) and AEC substrate"

P-H2AX - used for immunofluorescence. IgG isotype controls were used. Manufacturer validation "2 µg/ml of this antibody detected phosphorylated histone H2A.X in HeLa cells treated with 0.5 µM staurosporine for 4-6 hours" Cited: HMGB1 facilitates repair of mitochondrial DNA damage and extends the lifespan of mutant ataxin-1 knock-in mice. Ito H et al. EMBO Mol Med. 2015 Jan;7 (1):78-101. doi: 10.15252/emmm.201404392. PMID: 25510912

H3K9me3 - used for immunofluorescence. IgG isotype controls were used. Manufacturer validation "ab8898 staining H3K9me3 in mouse 3T3MEF by ICC/IF (Immunocytochemistry/immunofluorescence). Cells were fixed with PFA, permeabilized with CSK buffer and MeOH and blocked with 5% BSA for 30 minutes at 21°C. Samples were incubated with primary antibody (1/500 in PBS +5% BSA + 01% Tween20) for 2hours at 21°C. An undiluted Alexa Fluor® 546-conjugated Goat antirabbit. Cited: Heterochromatin loss as a determinant of progerin-induced DNA damage in Hutchinson-Gilford Progeria. Chojnowski A et al. Aging Cell. 2020 Mar;19 (3):e13108. doi: 10.1111/acel.13108. Epub 2020 Feb 22. PMID: 32087607 .

53BP1 - used for immunfluorescence. IgG isotype controls were used. Manufacturer validation "Knockout Validated: 53BP1 Antibody [NB100-304] - 53BP1 was detected in immersion fixed HeLa cells (left) but was not detected in 53BP1 knockout Hela cells (right) using Rabbit Anti-human 53BP1 polyclonal antibody (Catalog #NB100-304) at 0.3 ug/mL for 3 hours at room temperature. Cells were stained using the NorthernLights™ 557-conjugated Anti-Rabbit IgG Secondary Antibody (red; Catalog # NL004) and counterstained with DAPI (blue). Specific staining was localized to nuclei. Cited: DNA damage response inhibition at dysfunctional telomeres by modulation of telomeric DNA damage response RNAs. Rossiello F et al. Nat Commun. 2017. PMID: 28239143

SMA used for immunofluorescence of frozen tissue sections. IgG isotype controls were used. Manufacturer validation "IHC image of ab125057 staining in human breast ductal carcinoma formalin fixed paraffin embedded tissue section, performed on a Leica Bond system using the standard protocol B. The section was pre-treated using heat mediated antigen retrieval with sodium citrate buffer (pH6, epitope retrieval solution 1) for 20 mins. The section was then incubated with ab125057, 1µg/ml, for 15 mins at room temperature and detected using an HRP conjugated ABC system. DAB was used as the chromogen. The section was then counterstained with haematoxylin and mounted with DPX. Cited: Extensive Proliferation of a Subset of Differentiated, yet Plastic, Medial Vascular Smooth Muscle Cells Contributes to Neointimal Formation in Mouse Injury and Atherosclerosis Models. Chappell J et al. Circ Res. 2016 Dec 9;119(12):1313-1323. doi: 10.1161/CIRCRESAHA.116.309799. Epub 2016 Sep 28.

CD45 used for immunofluorescence of frozen tissue sections. IgG isotype controls were used. Manufacturer validation "Each lot of this antibody is quality control tested by immunofluorescent staining with flow cytometric analysis" Cited: Impaired immune surveillance accelerates accumulation of senescent cells and aging. Ovadya Y et al. Nat Commun. 2018 Dec 21;9(1):5435. doi: 10.1038/s41467-018-07825-3. PMID: 30575733

CD3 used for immunofluorescence of frozen tissue sections. IgG isotype controls were used. Manufacturer validation "Each lot of this antibody is quality control tested by immunofluorescent staining with flow cytometric analysis" Cited: Impaired immune surveillance accelerates accumulation of senescent cells and aging. Ovadya Y et al. Nat Commun. 2018 Dec 21;9(1):5435. doi: 10.1038/s41467-018-07825-3. PMID: 30575733

CD68 used for immunofluorescence of frozen tissue sections. IgG isotype controls were used. Manufacturer validation "Each lot of this antibody is quality control tested by intracellular immunofluorescent staining with flow cytometric analysis" Cited: Cited: Huntingtin is required for normal excitatory synapse development in cortical and striatal circuits. McKinstry SU et al. J Neurosci. 2014 Jul 9;34(28):9455-72. doi: 10.1523/JNEUROSCI.4699-13.2014. PMID: 25009276

VCAM-1 – used for immunofluorescence of frozen tissue sections. IgG controls were used. Validated on mouse spleen (data not shown). Manufacturer validation: "Each lot of this antibody is quality control tested by immunofluorescent staining with flow cytometric analysis." Cited: Expression, regulation, and function of atypical chemerin receptor CCRL2 on endothelial cells, Monnier et al, J Immuno, 2012, PMID: 22696441.

ICAM-1 - used for immunofluorescence of frozen tissue sections. IgG controls were used. Validated on mouse spleen (data not shown). Manufacturer validation: "Each lot of this antibody is quality control tested by immunofluorescent staining with flow cytometric analysis." Cited: Endothelial TGF-β signalling drives vascular inflammation and atherosclerosis, Chen et al, Nat Metab, 2019, PMID: 31572976

Eukaryotic cell lines

Policy information about cell lines

Cell line source(s)	Human VSMCs were obtained from patients undergoing coronary artery bypass/valve replacement respectively, under informed consent using protocols approved by the Cambridge or Huntingdon Research Ethical Committee
Authentication	The VSMC origin was confirmed by qPCR analysis for specific SMC markers

Mycoplasma contamination	All cell lines used in this study were routinely screened for mycoplasma contamination
Commonly misidentified lines (See ICLAC register)	N/A

Animals and other organisms

Policy information about studies involving animals; ARRIVE guidelines recommended for reporting animal research

Laboratory animals	Mice strain C57/Bl6. Myh-CreERt mice are Y chromosome-linked; therefore all studies were performed using males. All mice were used at the age of 13 weeks.
Wild animals	N/A
Field-collected samples	N/A
Ethics oversight	All animal experiments were performed under the Animals (Scientific Procedures) Act 1986 Amendment Regulations 2012 and were approved by Cambridge University Animal Welfare and Ethical Review Body (AWERB).

Note that full information on the approval of the study protocol must also be provided in the manuscript.
